# Genomic signatures of the evolution of defence against its natural enemies in the poisonous and medicinal plant *Datura stramonium* (Solanaceae)

**DOI:** 10.1038/s41598-020-79194-1

**Published:** 2021-01-13

**Authors:** I. M. De-la-Cruz, A. Hallab, U. Olivares-Pinto, R. Tapia-López, S. Velázquez-Márquez, D. Piñero, K. Oyama, B. Usadel, J. Núñez-Farfán

**Affiliations:** 1 Departamento de Ecología Evolutiva, Instituto de Ecología, Universidad Nacional Autónoma de México (UNAM), Mexico City, Mexico; 2 Escuela Nacional de Estudios Superiores, Universidad Nacional Autónoma de México (UNAM), Campus Juriquilla, Querétaro, Mexico; 3 Escuela Nacional de Estudios Superiores and Laboratorio Nacional de Análisis y Síntesis Ecológica (LANASE), Universidad Nacional Autónoma de México (UNAM), Campus Morelia, Morelia, Michoacán, Mexico; 4 IBG-4 Bioinformatics, CEPLAS, Forschungszentrum Jülich, Julich, Germany; 5 Institute for Biology I, RWTH Aachen University, Aachen, Germany

**Keywords:** Plant evolution, Computational biology and bioinformatics, Evolution, Genetics, Plant sciences

## Abstract

Tropane alkaloids and terpenoids are widely used in the medicine and pharmaceutic industry and evolved as chemical defenses against herbivores and pathogens in the annual herb *Datura stramonium* (Solanaceae). Here, we present the first draft genomes of two plants from contrasting environments of *D. stramonium*. Using these de novo assemblies, along with other previously published genomes from 11 Solanaceae species, we carried out comparative genomic analyses to provide insights on the genome evolution of *D. stramonium* within the Solanaceae family, and to elucidate adaptive genomic signatures to biotic and abiotic stresses in this plant. We also studied, in detail, the evolution of four genes of *D. stramonium*—Putrescine *N*-methyltransferase, Tropinone reductase I, Tropinone reductase II and Hyoscyamine-6S-dioxygenase—involved in the tropane alkaloid biosynthesis. Our analyses revealed that the genomes of *D. stramonium* show signatures of expansion, physicochemical divergence and/or positive selection on proteins related to the production of tropane alkaloids, terpenoids, and glycoalkaloids as well as on *R* defensive genes and other important proteins related with biotic and abiotic pressures such as defense against natural enemies and drought.

## Introduction

Plant species from the Solanaceae family, that includes numerous economically and ecologically important species (e.g., tomato, potato and tobacco) produce diverse secondary metabolites (tropane alkaloids, terpenoids and glycoalkaloids), that affect growth, development and/or survival of herbivore insects and pathogens (bacteria, fungi, virus)^1^. In particular, tropane alkaloids belong to the world’s oldest plant medicines used by humans, and these compounds are abundantly present in the Solanaceae family as well as in Erythroxylaceae, Convolvulaceae, Brassicaceae, and Euphorbiaceae families^1,2^. Within the Solanaceae family, the annual herb *Datura stramonium* produces the highest concentration of tropane alkaloids^2^. Scopolamine, atropine (hyoscyamine), and anisodamine are the main tropane alkaloids of *D. stramonium*^3^. Scopolamine and atropine historically have been used for asthma, rheumatism and as spasmolytic drugs^2,4^. In fact, scopolamine is one of the essential active medical compounds according to the World Health Organization (WHO)^5^.

In Mexico, plants of the genus *Datura*, known as *Toloache*, have been used by native cultures since pre-Columbian times^4,6^. The species of the genus *Datura *(Solanaceae) are native to dry, temperate, tropical and subtropical regions of North America, and occur mostly in Mexico, considered its center of origin^6,7^. *Datura stramonium*, although native to North America, has expanded its distribution, owing to humans, worldwide except to polar and subpolar climate zones^8^. This species occurs, distinctively, in human-disturbed habitats^8^.

Recent advances in DNA sequencing have allowed the genome assembly of several important model species from the Solanaceae family^9–17^. However, genome sequences of non-model species from this plant family are still scarce. Non-model species such as *D. stramonium* could be of wide interest because they offer new modes of investigating the ecological and evolutionary processes that plants face in their natural environments and how they respond to pollution, human disturbance and climate change^18^. Furthermore, the availability of genomes from non-model Solanaceae species would be of great value to better understand the evolution of this family^18^.

Here, we present the first draft genomes of two plants of *D. stramonium *that were selected from two contrasting populations of Mexico (Teotihuacán, State of Mexico and Ticumán, State of Morelos)^19,20^. Plants from Ticumán produce a higher concentration of tropane alkaloids than those from Teotihuacán^3^. Evidence points out that this differentiation is adaptive due to different herbivores pressures between populations^3,21^. In this study, we also carried out extensive comparative genomic analyses with a total of 13 Solanaceae species (including both genomes of *D. stramonium*) to explore the phylogenetic divergence of the Solanaceae family and to identify adaptive genomic signatures to biotic and abiotic stresses in the *D. stramonium* genome. Furthermore, we studied four key genes (Putrescine *N*-methyltransferase, Tropinone reductase I, Tropinone reductase II, Hyoscyamine-6S-dioxygenase) of *D. stramonium* that are involved in the biosynthesis of the tropane alkaloids^22^, and we relate this genetic information with the concentration of 19 tropane alkaloids that were quantified for the two genomes using Liquid chromatography-time-of-flight-mass spectrometry (HPCL-TOF-MS).

## Results and discussion

### Genome sequencing and assembly

**Figure 1:**
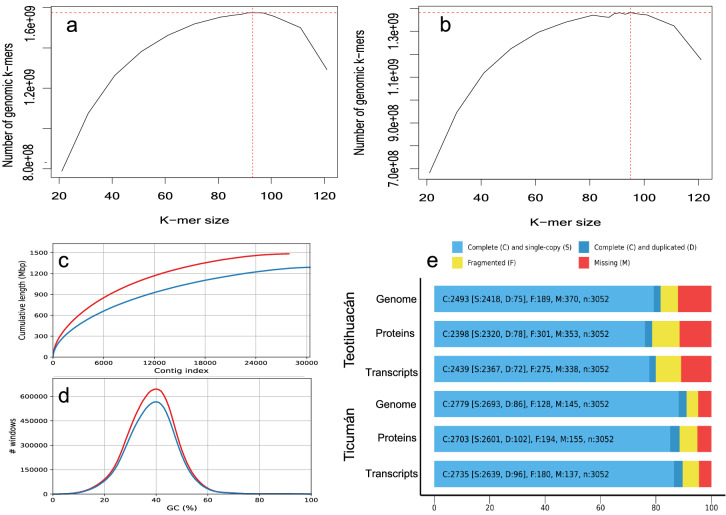
Genome size estimation in *Datura stramonium* by the K-mer distribution of the Illumina DNA reads (**a**) Ticumán, (**b**) Teotihuacán. (**c**) GC content plot shows the distribution of GC content in the contigs (red line = Ticumán, blue line = Teotihuacán). (**d**) Cumulative length plot shows the growth of contig lengths. On the x-axis, contigs are ordered from the largest to smallest. The y-axis gives the size of the x largest contigs in the assembly. This is the total genome assembled (red line = Ticumán, blue line = Teotihuacán). (**e**) BUSCO plots for the two *Datura stramonium* genomes, transcriptomes and proteomes predicted by MAKER program. The plot shows quantitative measures for the assessment of the genome completeness based on evolutionarily informed expectations of gene content from near-universal single-copy orthologs selected from the “Solanaceae odb10*” database. See Supplementary Table S3 online.

DNA was isolated and assembled from two diploid plants collected from two populations of *D. stramonium*; Ticumán State of Morelos, Mexico and Teotihuacán, State of Mexico, Mexico. 323M PE (paired-end) raw sequences (2 X 150b) were obtained from Illumina HiSeq 4000 sequencing; corresponding to 112 Gb and an average 30.85-fold genome coverage for the Ticumán individual, while 318M PE sequences corresponding to 110 Gb and 30.29-fold genome coverage were generated for the Teotihuacán individual (Supplementary Table S1 online). After trimming the PE sequences, we obtained 305M and 303M reads for Ticumán and Teotihuacán, respectively (Supplementary Table S1 online). For PacBio Sequel I sequencing, we obtained 9,995,713 subreads corresponding to 37 Gb for the Ticumán individual, while for Teotihuacán individual 9,505,413 subreads were generated, corresponding to 30 Gb (Supplementary Table S1 online). The frequency of K-mers estimated a genome size of 1.38 Gb for Teotihuacán and 1.57 Gb for Ticumán (Fig. 1a,b). However, the total length of the assembly was 1.28 Gb and 1.47 Gb for Teotihuacán and Ticumán, respectively (Table 1, Fig. 1c). Cell flow cytometry analysis did not indicate differences in genome sizes between both individuals, estimating a genome size of 1.7 Gb (Supplementary Table S2 online). Both assemblies showed a normal pattern for relative GC content (Fig. 1d). The total number of scaffolds was 27,915 and 30,392 for Ticumán and Teotihuacán genomes, respectively. Approximately, 1.05 Gb and 730 Mb of the Ticumán and Teotihuacán *Datura* genomes, respectively, showed contigs ≥ 50,000 bp (Table 1). N50 scaffold length resulted in 84,687- and 58,197-bp for Ticumán and Teotihuacán assembly, respectively (Table 1). Overall, the Ticumán genome was better assembled than the Teotihuacán genome (Table 1, Fig. 1c,d). This differentiation is because we obtained lower quality sequences from PacBio platform for the Teotihuacán plant.

**Table 1:** (a) Assembly statistics for both genomes of *Datura stramonium*. (b) Merqury quality values (QV). (c) Annotation statistics for the *Datura stramonium* genomes.

Assembly	Ticumán	Teotihuacán
**(a) Quast statistics**		
# contigs (≥ 0 bp)	27,915	30,392
# contigs (≥ 1000 bp)	27,843	30,344
# contigs (≥ 5000 bp)	26,349	29,471
# contigs (≥ 10,000 bp)	24,397	27,709
# contigs (≥ 25,000 bp)	17,380	17,390
# contigs (≥ 50,000 bp)	9343	7299
# contigs	27,900	30,385
Largest contig	3,131,142	2,112,153
Total length	1,482,568,706	1,288,884,002
GC (%)	38.47	38.45
N50	84,121	58,197
N75	44,455	32,640
L50	4557	5713
L75	10,641	13,166
# N's per 100 kbp	721.42	110.68
**(b) Merqury quality values**		
Genome completeness	88.27	77.98
QV	23.28	19.50
Error rate	0.004	0.01
**(c) Annotation statistics**		
Number of genes	33,856	30,934
Number of exons	176,756	163,107
Number of exons in cds	170,946	157,410
Number of introns in cds	137,090	126,476
Number of introns in exon	142,900	132,173
Mean mrnas per gene	1.0	1
Mean exons per mrna	5.2	5.3
Total gene length	147,987,251	132,043,897
% of genome covered by gene	16	14.2
% of genome covered by cds	2.5	2.6
% of genome covered by exon	2.7	2.9
% of genome covered by intron from cds	7.1	7.2
% of genome covered by intron from exon	7.3	7.4

Our *Datura* genomes covered 91% and 81.7% complete single copy orthologs (BUSCOs) for Ticumán and Teotihuacán, respectively, of the 3052 total BUSCOs searched (Supplementary Table S3 online, Fig. 1e). Predicted transcripts and proteins from MAKER for the Teotihuacán genome covered 80% and 78.6% (respectively) of BUSCOs, while for the Ticumán genome, transcripts and proteins covered 88.5% and 89.6% (respectively) of BUSCOs (Supplementary Table S3 online, Fig. 1e). Both *D. stramonium* genomes share 2233 and 2173 transcripts and proteins, respectively, of the 3052 total BUSCOs searched. Consensus quality values (QV) obtained from Merqury revealed a QV of 23.28 (error rate = 0.004) and 19.50 (error rate = 0.01) for the Ticumán and Teotihuacán genome, respectively (Table 1, Supplementary Fig. S1 online). Quality values above 20 corresponds to 99% of assembly consensus accuracy^23^. These results suggest that the Ticumán assembly has higher QV than the Teotihuacán assembly, but still both assemblies are similar^23^ (Table 1, Supplementary Fig. S1 online). Likewise, Merqury values of genome completeness were 88.27% and 77.98 for the Ticumán and Teotihuacán genome, respectively (Table 1). We also mapped the raw PE sequences from each individual to its corresponding assembly for genome validation quality, the overall mapping rates were 96.14% and 89.47% for Ticumán and Teotihuacán, respectively (Supplementary Table S4 online). Completeness of single copy orthologs, Merqury quality values and mapping rates indicate a good quality assembly for both genomes but especially for the Ticumán individual.

The alignment between the two genome assemblies revealed a total of 6,673,981 SNPs (Supplementary Table S5 online). The percentage average identity of 1-to-1 alignment blocks between the Ticumán and Teotihuacán genomes was 97.92 (Supplementary Table S5 online). However, since both genome assemblies are still fragmented, the structural variation investigated between both assemblies prevents the discovery of large-scale translocations and inversions^24^ (see Supplementary Table S5 online).

The MAKER annotation pipeline included 33,856 and 30,934 protein-coding genes (Table 1). The total genome covered by the genes for Teotihuacán was 14.2% and for Ticumán was 16% (Table 1). The total number of exons was 176,756 and 163,107 for Ticumán and Teotihuacán genomes, respectively (Table 1). The mean exons per mRNA was 5.2 for Ticumán and 5.3 for Teotihuacán. A total of 99% gene models showed high confidence matches (E-value ≤ 1e^−5^) in the UniProtKB/TrEMBL database. Other non-model Solanaceae species that have been sequenced with similar genome size of *D. stramonium* are *Petunia inflata* (genome size = 1.29 Gb) and *Petunia axilaris* (genome size = 1.26 Gb), and they were assembled in 83,639 and 136,283 scaffolds, respectively^24^. Our workflow using iteratively short and long sequences with moderate sequencing coverage to generate contigs and scaffolds revealed an accurate assembly^23^. PacBio sequences from Teotihuacán individual showed a higher error rate and this produced a shorter and more fragmented genome assembly than the Ticumán individual. This also affected the number of genes annotated. Nonetheless, this number in both genomes approximately is equal to the expected number in Solanaceae species. Furthermore, the percentage of missing BUSCOs was relatively low for both genomes, transcriptomes and proteomes^25^. Here, the number of complete BUSCOs for our genome assemblies, transcriptomes and proteomes is very similar to that reported for Tomato, Potato, Eggplant, Pepper, Tobacco and its wild relatives, as well as *P. inflata* and *P. axilaris*^9–11,13,14,17,26,27^.

### Repetitive landscape of *Datura* genomes

**Figure 2:**
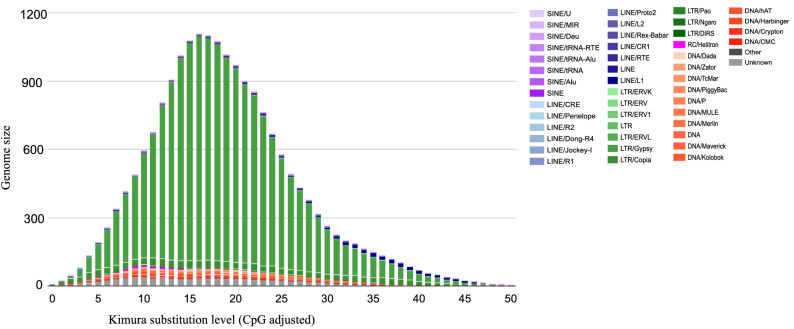
The repeat landscapes depict the relative abundance of repeat classes in the genome of *Datura stramonium* (Ticumán example) versus the Kimura divergence from the consensus. LTR/*Gypsy* family is the most represented repetitive element in the genome of *D. stramonium* (61.33%) followed by LTR *Copia* family. Genome of Teotihuacán also presents the same pattern.

*Datura* genomes are rich in repetitive DNA (as are most other plant genomes^28^). The repetitive landscape of our genomes revealed that 76.04% and 74.11% of the genomes are composed by repetitive elements (Supplementary Table S6 online, Fig. 2). These results reveal a higher proportion of repetitive elements than in other Solanaceae genomes, such as tomato, potato and *Petunia* species, and nearly similar to the repetitive landscapes of *Nicotiana* and *Capsicum* genomes^9,10,14,26,27^ (Supplementary Table S7 online). Long terminal repeats (LTR) elements are the most abundant in the *D. stramonium* genomes (Supplementary Table S6 online, Fig. 2), covering 65.88% and 63.41% of the genomes for Ticumán an Teotihuacán, respectively (Supplementary Table S6 online, Fig. 2). The *Gypsy* family is the most LTR represented in both genomes covering 61.33% and 58. 71% for Ticumán and Teotihuacán genomes, respectively (Fig. 2). The *Copia* family represents almost the rest of the repetitive landscape for both genomes (Fig. 2). An analysis of the history of repetitive elements between *Nicotiana* and *Solanum* species revealed that all *Nicotiana* species experienced a recent independent wave of *Gypsy* retrotransposon expansion^12,26^ and this seems to have happened also in the *Datura* species.

### Comparative genomic analyses

**Figure 3:**
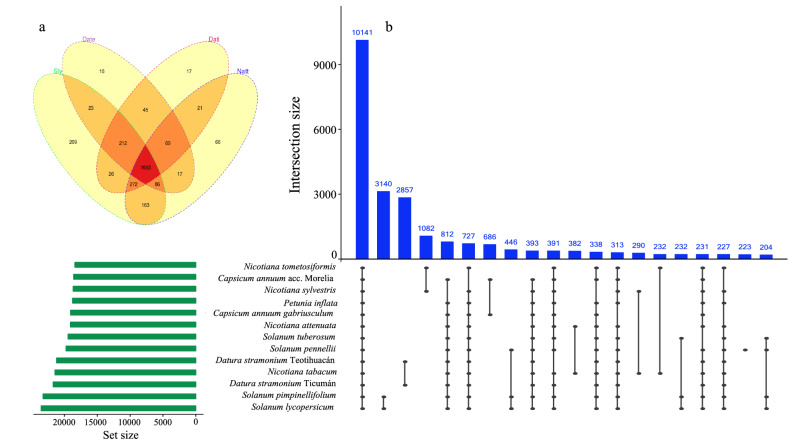
(**a**) Venn diagram shows 7653 InterProscan domains that are shared between *Datura stramonium* Teotihuacán (Date), *Solanum lycopersicum* (Sly), *Datura stramonium* Ticumán (Dati) and *Nicotiana attenuata* (Natt). 15 and 17 domains are exclusive for *D. stramonium* Teotihuacán and Ticumán, respectively. The UpSet plot shows the intersections of the set of orthogroups from the thirteen Solanaceae genomes. Each column corresponds to an orthogroup, and each row corresponds to one segment in a Venn diagram. Cells are either empty (grey-black), indicating that this set is not part of that intersection, or filled, showing that the set is participating in the intersection and that the species share that orthogroup. OrthoFinder assigned 480,594 genes out of 536,483 (89.6% of total) to 35,458 orthogroups or gene families. *Solanum pimpinellifolium* and *Solanum lycopersicum* were the species-pair with more orthogroups shared (3140), followed by the pair between *Datura stramonium* Ticumán and Teotihuacán (2857 shared orthogroups).

Protein coding genes from 11 genomes were sourced from the Sol Genomics Network (https://solgenomics.net/, see “Materials and Methods” section). We used these genomes along with both *D. stramonium* genomes to construct orthogroups (gene families) using OrthoFinder v2.3.3^29^. This program assigned 480,594 genes out of 536,483 (89.6% of total) to 35,458 orthogroups or protein families (Supplementary Table S8 online). Mean gene family size is 13.6 proteins, while fifty percent of all proteins were in proteins families with 19 or more proteins (G50 = 19) (Supplementary Table S8 online). There were 10,141 protein families with all species present (Fig. 3) and 181 of these consisted entirely of single-copy genes. The two species which shared the most protein families were *S. pimpinellifolium* and *S. lycopersicum* (Fig. 3).

**Figure 4:**
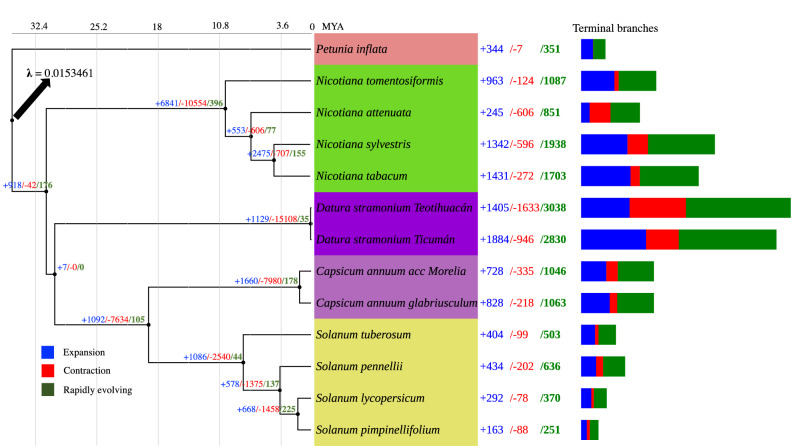
The species phylogeny shows four clades, the group of *Nicotiana* species, the clade of *Datura*, the group of *Capsicum* species and the *Solanum* group. *Petunia inflata* was selected as the outgroup species. *P. inflata* diverged from all the Solanaceae species studied here approximately 35 Mya. *Nicotiana* species diverged almost 32 Mya. While *Datura stramonium* diverged ~ 30.1 Mya from *Solanum*, *Capsicum* and *Nicotiana* species. The rate of gene gain and lost (λ) resulted from CAFE analysis was 0.0153461 for the whole tree. The terminal branch with the most rapidly significant evolving gene families is the one leading to *D. stramonium* clade. The internal branch with the largest numbers of significant contractions corresponds also to the most recent common ancestor of *Datura* species clade. The terminal branches with the most contractions are the one leading to *D. stramonium* Teotihuacán*.* While *D. stramonium* Ticumán showed the highest number of gene family expansions.

The species phylogeny shows four mayor clades, the group of *Nicotiana* species, the clade of *Datura*, the group of *Capsicum* species and the *Solanum* group. *Petunia inflata* was selected as the outgroup species (Fig. 4). *Petunia inflata* diverged from all the Solanaceae species studied here approximately 35 Mya, while *D. stramonium* diverged ~ 30.1 Mya from *Solanum*, *Capsicum* and *Nicotiana* species (Fig. 4). The divergence dates reported here are consistent with other phylogenies reported for the Solanaceae species^14,30^. The rate of gene gain and lost (λ) resulted from CAFE analysis was 0.015 for the whole tree (Fig. 4). The internal branch with the largest numbers of significant rapidly evolving gene families corresponds to the most recent common ancestor of *N. tomentosiformis* (Fig. 4). The terminal branch with the most rapidly significant evolving gene families is the one leading to *D. stramonium* clade (Fig. 4). The internal branch with the largest numbers of significant contractions corresponds also to the most recent common ancestor of *Datura* species clade (Fig. 4). The terminal branches with the most contractions are the one leading to *D. stramonium* Teotihuacán*.* While *P. inflata* is the species with the least contractions (Fig. 4). The internal branch with the largest significant number of expansions corresponds to the most recent common ancestor of *N. tomentosiformi*s. *Datura stramonium* Ticumán showed the highest number of gene family expansions (Fig. 4).

### Enrichment tests

We found 49 InterPro enriched domains in proteins subject to physicochemical divergence in both genomes (*p* < 0.01) (Supplementary Table S9 online). 94 enriched InterPro domains were detected in proteins with signal of expansion in both genomes (*p* < 0.01) (Supplementary Table S10 online). 56 enriched InterPro domains were detected in *Datura* proteins with positively selected conserved amino acids (codons) (Supplementary Table S11 online). Likewise, we found with the MapMan4 annotation a total of 14 enriched proteins with signal of expansion (Supplementary Table S12 online), 23 proteins with positively selected conserved amino acids (Supplementary Table S13 online), and 54 enriched proteins with physicochemical divergence (Supplementary Table S14 online). We found that either over-represented domains (enrichment test using InterproScan database) as well as over-represented proteins (enrichment test using MapMan4 database) with signal of expansion, positive selection or physicochemical divergence are related with immunity and defence against pathogens, virus, fungi and insect herbivores as well as related with responses to abiotic stresses such as drought and nutrients deficiency (Tables 2, 3, Supplementary Table S14 online).

**Table 2:** Classifications of domains related with defence against natural enemies (pathogens, viruses, fungi, oomycete, herbivores) subject to expansion, positive selection or physicochemical divergence. Some domains were detected to be expanded and positively selected.

Defensive domains	InterProscan ID	*p* value	Analysis
Virus X resistance protein-like	IPR038005	2.851E−13/1.120E−17	Ex, PS
Rx, N-terminal	IPR041118	3.412E−04/4.928E−09	Ex, PS
NB-ARC	IPR002182	9.735E−10/5.184E−21	Ex, PS
Actin family	IPR004000	7.157E−09	Ex
Actin, conserved site	IPR004001	9.950E−06/6.619E−07	Ex, PS
Actin/actin-like conserved site	IPR020902	2.980E−11/4.336E−04	Ex, PS
START-like domain superfamily	IPR023393	3.878E−04	Ex
Bet v I/Major latex protein	IPR000916	4.277E−08/9.020E−04	Ex, PS
Zinc finger, PMZ-type	IPR006564	4.402E−04	Ex
Late blight resistance protein R1	IPR021929	5.064E−05	Ex
Leucine-rich repeat domain superfamily	IPR032675	1.166E−05/4.319E−18	Ex, PS
Ribonuclease H-like superfamily	IPR012337	1.000E−04	PS
DNA-binding pseudobarrel domain superfamily	IPR015300	2.277E−03	Ex
Syntaxin, N-terminal domain	IPR006011	1.009E−03	FQ
Target SNARE coiled-coil homology domain	IPR000727	7.302E−03	FQ
Syntaxin/epimorphin, conserved site	IPR006012	1.041E−03	FQ
Transmembrane protein 131-like	IPR039877	1.041E−03	FQ
Ribonuclease II/R	IPR001900	7.031E−03	FQ
BRCA1-associated	IPR031099	9.441E−03	FQ
Trichome birefringence-like 45	IPR029981	4.774E−03	FQ

*p* value is showed for each analysis. The entire list for each analysis is showed in Supplementary Tables S9, S10 and S11.

*Ex* expanded, *PS* positive selected, *FQ* physicochemical divergence.

**Table 3:** Classifications of domains related with the biosynthesis of secondary compounds and that act as defence against natural enemies (pathogens, viruses, fungi, oomycete, herbivores) subject to expansion, positive selection or physicochemical divergence. Some domains were detected to be expanded and positively selected.

Domains related with the biosynthesis of secondary compounds	InterproScan	*p* value	Pathway of secondary compounds	Analysis
Cytochrome P450 superfamily	IPR036396	7.888E−03/2.134E−10	Tropane, terpenoid	Ex, PS
Cytochrome P450, E-class, group I	IPR002401	4.539E−03/4.047E−12	Tropane, terpenoid	Ex, PS
Cytochrome P450	IPR001128	5.179E−03/1.033E−10	Tropane, terpenoid	Ex, PS
Cytochrome P450, conserved site	IPR017972	1.410E−08	Tropane, terpenoid	PS
Aminotransferase-like, plant mobile	IPR019557	8.030E−11	Tropane	Ex
Transferase	IPR003480	8.572E−05/6.310E−04	Tropane	Ex, PS
Isoprenoid synthase domain superfamily	IPR008949	1.909E−04	Isoprenoid	Ex
Terpene synthase, N-terminal domain	IPR001906	1.704E−05/2.295E−07	Terpenoid	Ex, PS
Terpene synthase, N-terminal domain	IPR036965	1.364E−04/3.426E−07	Terpenoid	Ex, PS
Terpenoid cyclases/protein prenyltransferase alpha-alpha toroid	IPR008930	1.140E−03/2.163E−07	Terpenoid	Ex, PS
Terpene synthase, metal-binding domain	IPR005630	5.064E−05/2.289E−06	Terpenoid	Ex, PS
Terpene cyclase-like 1, C-terminal	IPR034741	8.806E−03/2.533E−06	Terpenoid	Ex, PS
NADH:ubiquinone oxidoreductase	IPR003918	7.056E−03	Tropane	Ex
NADH-quinone oxidoreductase, subunit D superfamily	IPR038290	1.571E−04	Tropane	Ex
NADH-quinone oxidoreductase, subunit D	IPR001135	8.570E−05	Tropane	Ex
NADH-quinone oxidoreductase chain 4	IPR022997	8.806E−03	Tropane	Ex
Glutathione S-transferase, C-terminal-like	IPR010987	3.430E−07	Glutathione	PS
Glutathione Transferase family	IPR040079	4.542E−04	Glutathione	PS
Glutathione S-transferase, N-terminal	IPR004045	9.960E−07	Glutathione	PS
Glutathione S-transferase, C-terminal	IPR004046	1.970E−05	Glutathione	PS
Phosphoethanolamine *N*-methyltransferase	IPR025771	4.280E−07	Tropane	FQ
Glycoside hydrolase, family 35	IPR001944	4.176E−04	Tropane, terpenoid, glycoside, isoprenoid	FQ
Glycoside hydrolase 35, catalytic domain	IPR031330	3.637E−04	Tropane, terpenoid, glycoside, isoprenoid	FQ
Glycosyltransferase family 92	IPR008166	2.379E−03	Tropane, terpenoid, glycoside, isoprenoid	FQ
Glycosyl transferase, family 31	IPR002659	7.031E−03	Tropane, terpenoid, glycoside, isoprenoid	FQ
NADPH-cytochrome P450 reductase	IPR023208	4.774E−03	Tropane, terpenoid	FQ
SNF1-related protein kinase regulatory subunit beta-2	IPR030070	4.740E−06	Terpenoid	FQ
Association with the SNF1 complex (ASC) domain	IPR006828	2.360E−05	Terpenoid	FQ

*p* value is showed for each analysis. The entire list for each analysis is showed in Supplementary Tables S9, S10 and S11.

*Ex* expanded, *PS* positive selected, *FQ* physicochemical divergence.

### Domains associated with defensive proteins (*R* genes)

Several domains have been associated as fundamental components of the *R* genes in *D. stramonium* genomes (Table 2) but some notable domains in expanded and positively selected proteins were found in *D. stramonium* (Table 2); the Virus X resistance protein-like (IPR038005) and Rx, N-terminal (IPR041118) are domains that confer resistance against the potato virus X^31,32^. IPR038005 domain has been identified in a family of resistance proteins that recognize pathogen effector proteins and trigger a response that may be as severe as localized cell death^32^. The NB-ARC (IPR002182) and Leucine-rich repeat (LRR) domain superfamily (IPR032675) (Table 2), interact and release a signal to initiate an event of immunity against pathogens^31,33^.

Resistance to a diverse range of pathogens, including nematodes, fungi, bacteria, and viruses involves LRR proteins either as resistance proteins or as proteins required for resistance proteins to function^34^. We found that the LRR-XII kinase and SD-1 kinase proteins had positively selected codons in *Datura* genomes (Supplementary Table S13 online). Magalhães et al.^35^ found a large expansion of LRR-XII in *Citrus* genomes, suggesting that it might play a key role in adaptive responses in host–pathogen co-evolution, related to the perennial life cycle and domestication of the citrus crop species. Likewise, it has demonstrated that SD-1 kinase protein is a plant receptor with roles in signaling and plant defense^36^. Moreover, we found several proteins belonging to the Kinase superfamily with significant physicochemical divergence (Supplementary Table S14 online). Kinase superfamily proteins have been related with different stresses including pathogen invasion^37^.

The Late blight resistance domain R1 (IPR021929) (Table 2) showed a significant expansion signal. The R1 is a protein for resistance to late blight, the most destructive disease in potato cultivation worldwide^38^. On the other hand, the Trichome birefringence-like 45 (IPR029981) domain had physicochemical divergence signal (Table 2). This domain is involved in non-host resistance (NHR) or plant immunity to non-adapted pathogen species^39^.

### Domains and proteins related with the biosynthesis of Terpenoids

We found in both enrichment analyses (InterPro and MapMan4 annotation) proteins with signal of expansion, physicochemical divergence and positively selected that are directly related with the biosynthesis of terpenoids (Table 3, Supplementary Tables S9-S14 online). For instance, Cytochrome P450 domain is related in the biosynthesis of terpenoids and has been associated as a key domain in the production of different terpenoids that mediate plant defence against herbivores^40^. We also found domains directly related in the biosynthesis of terpenoids in *D. stramonium* protein families with significant expansion and with positively selected codons. These families comprise Terpene synthases with the N-terminal domain (IPR001906), Terpenoid cyclases/protein prenyltransferase alpha-alpha toroid (IPR008930), Terpene synthase, metal-binding domain (IPR005630) and Terpene cyclase-like 1, C-terminal (IPR034741) (Table 3). Enrichment analysis with MapMan4 revealed that mono/sesquiterpene/diterpene synthase family proteins showed signal of expansion and with positively selected codons (Supplementary Tables S12, S13 online). Several studies have indicated that terpene synthases are the primary enzymes in the formation of terpene metabolites^41^. It has reported that terpene compounds can act as defense metabolites against herbivores or they also play a role as attractants to carnivorous arthropods that prey upon or parasitise herbivores, and so reduce further damage^41–44^. A recent study carried out with experimental populations of *D. stramonium* has identified a triterpenoid compound involved as defense against the most dangerous herbivore of this species, the larvae of *Lema daturaphila* (Chrysomelidae)^21^. Also, several terpenoids have been reported to mediate plant-plant communication^45^.

### Enriched proteins related with abiotic stresses

A notable domain, the SNF1-related protein kinase regulatory subunit beta-2 (IPR030070) was detected in proteins with physicochemical divergence (Supplementary Tables S9, S15 online). This domain has been implicated in the response against drought, and in the efficiency of carbohydrate metabolism and in the response to glucose limitation^46,47^. Also, we found over-represented domains in proteins with signal of expansion and with positively selected codons containing Galactose oxidase/kelch, beta-propeller (IPR011043) domain (Supplementary Table S15 online). This domain is also involved in the stress responses induced under Fe deficiency in the roots and also related as defence protein^48^. Kinase proteins showed signal of physicochemical divergence (Supplementary Table S15 online), and these proteins have been related with different abiotic stresses including light, temperature, and nutrient deprivation^35^.

### Genes involved in the tropane alkaloid biosynthesis

Notable domains involved in the tropane alkaloids pathway were proteins of families expanded in the *Datura* branch and with positively selected conserved amino acids (codons) (Supplementary Tables S10, S11 online). For instance, Cytochrome P450 (IPR001128), Transferase (IPR003480), NADH:ubiquinone oxidoreductase (IPR003918) and Phosphoethanolamine *N*-methyltransferase (IPR025771) (Table 3). Cytochrome P450 is involved in the rearrangement of Littorine (a kind of tropane alkaloid) to produce atropine/hyosciamine and scopolamine^49,50^. This step is very important in the biosynthesis of scopolamine via the Hyoscyamine (6S)-dioxygenase gene (*h6h*)^49,50^. Indeed, the enzymes that participate in the tropane alkaloid biosynthesis belong to the classes of oxidoreductases and transferases^22^, such as we detected in enriched proteins with signal of expansion, positively selected and proteins with physicochemical divergence (Table 3).

**Figure 5:**
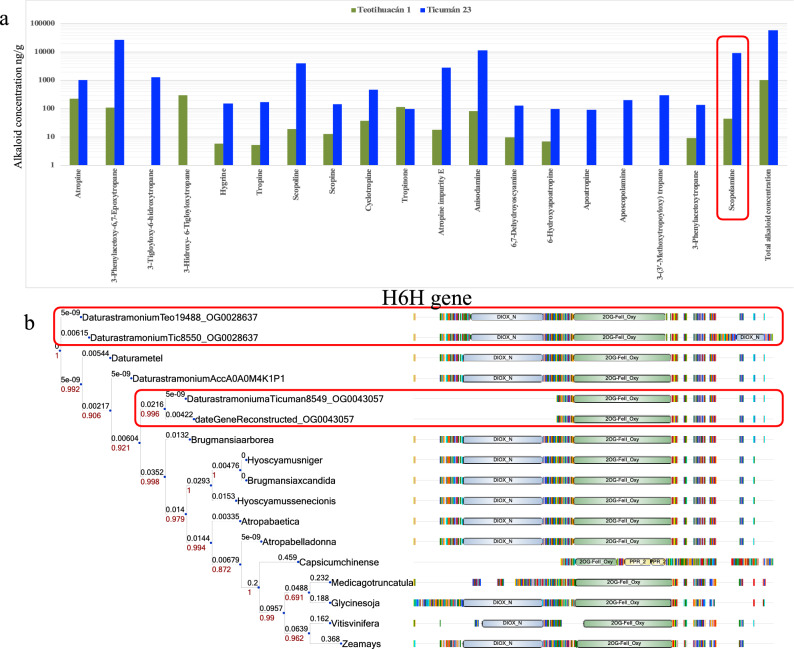
(**a**) Tropane alkaloid differentiation between Ticumán and Teotihuacán plants (Log scale) that were used to sequence their genomes. Almost all tropane alkaloids showed higher concentration in Ticumán than the Teotihuacán plant. Scopolamine alkaloid is highlighted with a red box. (**b**) *h6h* gene phylogeny was generated using 17 genes. Results revealed that two copies of *h6h* are found in both genomes of *D. stramonium* (highlighted with a red box). These two gene copies were distributed in two different gene families (OG0028637 and OG0043057). The name of the family was added at the end of the gene name. The gene DaturastramoniumTic8550_OG0028637 (Ticumán genome) have two domains of DIOX_N (PF14226), while all its homologous of the phylogeny only have one DIOX_N domain. The higher production of scopolamine alkaloid in the Ticumán genome could be related with this additional domain and different architecture in the Ticumán *h6h* gene.

Within tropane alkaloids, the *pmt* gene family showed significant gene expansion during the evolution of the *Datura* genus; the last common ancestor of *D. stramonium* had only one gene (Supplementary Table S16 online), while *D. stramonium* Ticumán and Teotihuacán have three and two gene copies, respectively (Supplementary material S16 online). Pfam annotation of *pmt* genes showed that the gene dati7568, which belongs to the Ticumán genome, has an extra domain of spermine-synthase in comparison with its homolog from Teotihuacán (Supplementary Fig. S2 online). Kasukabe et al.^51^ found that the overexpression of spermidine-synthase enhanced tolerance to multiple environmental stresses including herbivory and pathogenesis. Moreover, *pmt* is the key gene catalyzing the formation of *N*-methylputrescine from putrescine and S-adenosyl-L-methionine and this enzyme triggers the production of hygrine and other different tropane alkaloids^22^. HPLC-TOF-MS results revealed that the plant of Ticumán showed 26.63-fold of hygrine concentration than the plant of Teotihuacán (Supplementary Table S17 online, Fig. 5 a). In fact, a differentiation of 59-fold was obtained in total tropane alkaloid concentration between Ticumán and Teotihuacán (Supplementary Table S17 online, Fig. 5a). Thus, it is possible that the additional domain of spermine-synthase confers overproduction of tropane alkaloids in the Ticumán genome.

**Figure 6:**
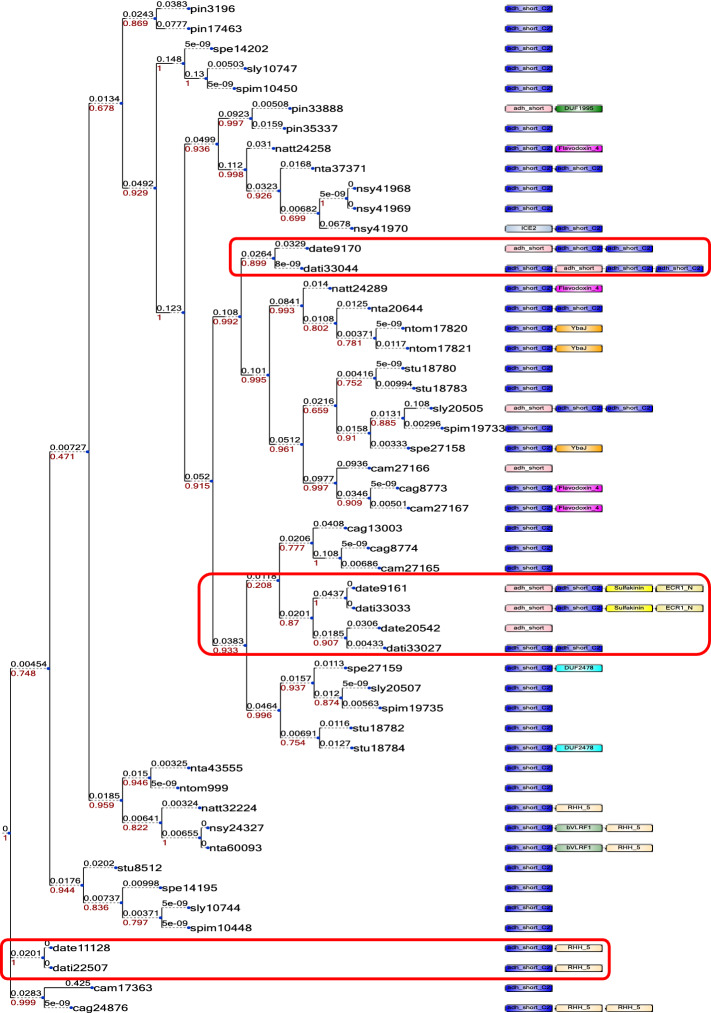
Four copies of the *tpr* I gene for both genomes were observed (highlighted with a red box). The gene date9161 (Teotihuacán) and dati33033 (Ticumán) have two different domains compared with the rest of the *tpr* I genes of *Datura*; Sulfakinin (PF08257) and ECR1_N domain (PF14382). Different architecture is showed for the *tpr* I genes, date9170 and dati33044. However, the gene dati33044 (Ticumán) have one additional domain of adh_short_C2 (PF13561), in comparison with its homologous date9170 (Teotihuacán). Likewise, dati33027 showed and additional adh_short_C2 domain in comparison with its closer homologous date20542. The Ticumán plant produced 31.95-fold tropine concentration than Teotihuacán. This chemical differentiation in tropine concentration could be related with this additional domain (adh_short_C2) that was observed in the Ticumán *tpr* I genes mentioned above. Another gene copies of *tpr* I, date11128 (Teotihuacán) and dati22507 (Ticumán) have the domain, RHH_5 (PF07878), this domain has been described as a toxin–antitoxin system (TA).

No expansion was detected for *tpr* I gene in *D. stramonium* (Supplementary Table S16 online). However, we observed four copies of this gene in both *Datura* genomes (Fig. 6). The gene date9161 (Teotihuacán) and dati33033 (Ticumán) have two domains absent in the other *tpr* I *Datura* genes; Sulfakinin (PF08257) and ECR1_N domain (PF14382) (Fig. 6). Wei et al.^52^ found that the sulfakinins may reduce the sensitivity of taste receptor of *Schistocerca gregaria* (Acrididae). Here, we found that sulfakinin domain is observed in *D. stramonium* but not in the other Solanaceae species (Fig. 6). Likewise, ECR1_N is an N-terminal region of the exosome complex of resistance proteins^33^. The *Datura tpr* I genes date9170 and dati33044 had a domain architecture different from the *tpr* genes of the other studied Solanaceae (Fig. 6). However, the gene dati33044 (Ticumán) has one additional domain adh_short_C2 (PF13561), in comparison with its homolog date9170 (Teotihuacán) (Fig. 6). Likewise, dati33027 showed an additional adh_short_C2 domain in comparison with its homolog date20542 (Fig. 6). We observed that the Ticumán plant produced *ca*. 32 times more tropine than the Teotihuacán plant (Supplementary Table S17 online, Fig. 5 a). This chemical difference in tropine concentration could be related with this additional domain (adh_short_C2) that was observed in the Ticumán genome. It has reported that the domain adh_short_C2 plays a role as anti-microbial and anti-parasitic molecules^53^.

Other gene copies of *tpr* I, date11128 (Teotihuacán) and dati22507 (Ticumán) have the domain RHH_5 (PF07878) (Fig. 6), this domain has been described as a toxin-antitoxin system (TA)^54^. Toxin- antitoxin genes are often inherited through horizontal gene transfer and are associated with pathogenic bacteria^55^. TA systems are numerous in many plant-associated bacteria, but very little is known regarding their function and distribution in phytopathogens^54^. This TA system is also found in *N. attenuata*, *N. tabacum*, *N. sylvestris* and *C. annuum gabriusculum* (Fig. 6). Several studies about the production of secondary metabolites as defence against natural enemies have been reported for these species^1^.

Evidence of expansion for the gene family *tpr* II was detected (Supplementary Table S16 online). The analysis revealed that the last common ancestor of *D. stramonium* had one *tpr* II gene, while five and three copies of *tpr* II genes showed to be expanded from Teotihuacán and Ticumán, respectively (Supplementary Table S16 online). An interesting domain, (PapA_C) is present in the gene copy date754 (Supplementary Fig. S3 online). This domain is so-called Polyketide- associated protein (Pap) that belongs to the subfamily of acyltransferases and has been found to be involved in the biosynthesis of secondary metabolites^53^. A Cobalamin (CobU) domain is found in the *Datura* gene dati23799 (Supplementary Fig. S3 online). Vascular plants neither synthesize nor require vitamin B12 because they contain cobalamin-independent methionine synthase (MetE)^56^. However, herbivores have been found to obtain their dietary quota of cobalamin from plants contaminated with cobalamin-producing soil bacteria (rhizobia) that grow in roots and nodules of plants^56^. Thus, more studies are needed to prove and assess the interaction between mycorrhizal and *D. stramonium*.

Hyoscyamine (6S)-dioxygenase gene (*h6h*) is the last rate-limiting enzyme directly catalyzing the formation of atropine and scopolamine in tropane alkaloids biosynthesis pathway^22^. As we have pointed out, scopolamine is the main secondary metabolite of *D. stramonium* with pharmaceutic and medical interest^57^. First, our results revealed that two copies of *h6h* are found in both genomes of *D. stramonium* (Fig. 5b). However, these copies were distributed in two different gene families (gene families “OG0028637” and “OG0043057”) (Fig. 5b). Indeed, both gene families were composed by only two genes; one from the Ticumán plant and one from the Teotihuacán plant. Therefore, we used 17 *h6h* genes (13 genes retrieved from Uniprot database and four from our *D. stramonium* genomes, see “Materials and Methods” section) to construct an artificial gene family. We carried out a multiple sequence alignment and reconstruct the phylogeny. Also, using the Pfam database the protein domain architecture of these genes was identified. The *h6h* copy gene of *D. stramonium* (DaturastramoniumTic8550_OG0028637) from Ticumán has two domains of DIOX_N (PF14226), while the above homologs have only one DIOX_N domain (Fig. 5b). In contrast, only three genes in this *h6h* family are composed of a single domain (2OG-FeII_Oxy, PF03171); two of them belong to our *D. stramonium* genomes and one are found in the *Medicago truncatula* genome (Fig. 5b). The DIOX_N domain is composed of a highly conserved N-terminal region of proteins with Oxoglutarate/iron-dependent dioxygenase activity (2OG-FeII_Oxy)^57^. This extra domain (DIOX_N) observed in one *h6h* gene from the Ticuman plant (DaturastramoniumTic8550_OG0028637) could be involved in the higher production of atropine, anisodamine and scopolamine than the plant from Teotihuacán. (Supplementary Table S16 online, Fig. 5a,b).

In summary, the Ticumán genome showed 57.31-fold of total tropane alkaloids than the Teotihuacán genome. Differences in several tropane alkaloid concentrations between Ticumán and Teotihuacán seem to be related with the differences in domain architecture of the four genes studied here involved in the tropane alkaloid biosynthesis. However, these results must be confirmed with expression data and/or qPCR of these important genes. Different evidence has pointed out that tropane alkaloids are implicated in resistance against herbivores in *D. stramonium*^21,58–60^ and that the selective value of tropane alkaloids preventing or reducing herbivory varies among populations of this plant species, depending on the type of enemies (specialist or generalists herbivores, fungi, pathogens, oomycete)^21,58–60^.

## Conclusions

The information generated here will provide support for future studies in the non-model species *D. stramonium*. Understanding the evolution, adaptation and the ecological role of tropane alkaloids and other secondary metabolites such terpenoids is necessary to disentangle its role in defence against natural enemies. We described how the *D. stramonium* genome expanded and we detected positive selection and physicochemical divergence on terpenoids, tropanes, glycosides, *R* genes, and proteins related with abiotic stresses such as drought. Indeed, the availability of these draft genomes provides a tool for future studies to better understand the genome evolution of the Solanaceae family and for other scientific fields such as medicine.

## Materials and methods

### Selection of the parent genomes

The two selected genomes were extracted from two different populations, Ticumán in the State of Morelos, 18° 45′ 39.90" N, 99° 7′ 13.86" W, and Teotihuacán, in the State of Mexico, 19° 41′ 6.96" N, 98° 52′ 19.63" W^3,19^. We analyzed 21 tropane alkaloids via HPLC-TOF-MS from 47 (Ticumán) and 45 (Teotihuacán) plants under controlled conditions (green house experiment) and we selected these two individuals that were the most differentiated in their total tropane alkaloid concentration (Teotihuacán = 1018 ng/g; Ticumán = 59,051 ng/g). Chemical conditions and details of samples extraction and mass spectrometry analyses can be consulted in De-la-Cruz et al.^3^.

### DNA extraction, genomic library preparation and sequencing

To obtain a high-quality de novo assembly, we combined data generated from short insert paired- end libraries from Illumina sequencing, with long read sequencing by PacBio Sequel I sequencing. First, gDNA was extracted from the two individuals. gDNA was isolated from fresh leaves with a modified CTAB mini-prep protocol^61^. The total amount of gDNA was measured using Qubit dsDNA HS Assay Kit (Invitrogen, Thermo Fisher Scientific, Waltham, USA). A total of 200 ng of gDNA were used for library preparation. Libraries were sheared on the Covaris M220 Focused-ultrasonicator (Covaris Massachusetts, USA) then prepped for 150PE (paired-end) Illumina HiSeq 4000 sequencing, using the Kapa Hyper prep Illumina library prep kits. Final libraries were visualized on the Agilent Fragment Analyzer, then quantified and pooled equimolar with Kapa qPCR Illumina library quant Universal Kits. Demultiplexing was then performed with the Illumina bcl2fastq v2.19 software (free available at: https://support.illumina.com/downloads/bcl2fastq-conversion-software-v2-20.html) and returned in fastq format.

The PacBio Sequel I sequencing (Pacific Biosciences) was performed by taking 20 ug of gDNA into SMRTbell library preparation for long-insert libraries with the PacBio Express Template preparation kit, followed by size selection at 15 kb on the Sage BluPippin. Libraries were then run on 10-h movies (length of time to continuously run the sequencing reaction) on a PacBio Sequel I using v2.0 chemistry. The sequencing and libraries preparation for both sequencing platforms was carried out in the QB3 Functional Genomics and Vincent J. Coates Sequencing Laboratories at the University of California, Berkeley.

### Preprocessing of sequenced short reads

Reads quality has a major impact on the quality of the resulting assembly, and the use of error-corrected reads increases the size of the contigs^62^. Illumina paired-end reads were trimmed using a phred quality score > 30 in TRIMMOMATIC v0.32^63^, following a sliding window trimming approach. We verified visually the quality (including contamination with Illumina paired-end adaptors) before and after trimming using the program FastQC^64^. This allowed us to only keep high-quality reads prior to the assembly steps.

### Genome size estimation

Quality trimmed Illumina paired-end sequences were used to estimate the genome size using KmerGenie v1.7016^65^. The best k-mer sizes were 95 and 93 for Teotihuacán and Ticumán, respectively. Also, the genome size was estimated through cell flow cytometry for both individuals carried out at National Laboratory of Flow Cytometry of the National Autonomous University of Mexico. To estimate the genome size, *Arabidopsis* col-1 ecotype and human PBMCs (male donor) were used as a reference. The nuclear DNA content of the sample was calculated with the formula:

$$Value\; 2C\; sample \left( {pg} \right) = Value\; 2C \;reference \times \frac{IMF\; sample}{{IMF\; reference }}$$

where *pg* is picograms and IMF is average fluorescence intensity.

### De novo genome assembly

Each individual of *D. stramonium* was assembled independently de novo*.* We followed the workflow of Chakraborty et al.^66^ for both plants, with modifications^66^. First, PacBio raw subreads (in bam format) were transformed to fasta format and assembled with Canu v1.8 pipeline that includes three stages: correction, trimming, and assembly^67^. PacBio only assembles of high error, long molecule sequences, depend upon redundancy between the various low-quality reads to ‘vote out’ errors and identify the true sequence in the sequenced individual^66^. Therefore, we used also a hybrid assembly approach suggested by Ye et al.^68^. For this, Illumina short reads were used to perform De Bruijn graph assembly with SparseAssembler^68^. The generated contigs from SparseAssembler were used with PacBio raw sequences to carry out a hybrid assembly using the program DBG2OLC^68^. An advantage of DBG2OLC program is that uses multiple sequence alignment to clean the PacBio reads and remove reads with structural errors (the so-called chimeras)^68^.

The program MUMmer v3^24^ was used to run the NUCmer wrap and the program delta-filter to compute unique alignments between the contigs from the Hybrid assembly (DBG2OLC) and PacBio assembly (Canu). DBG2OLC assembly was used as reference and Canu assembly was used as query. This last step allowed us to merge both assemblies (DBG2OC and Canu) using the program Quickmerge^66^. As the two assemblies used for merging come from the same genome, gaps in one assembly can be bridged using the corresponding sequences from the other assembly^66^. Thus, Quickmerge program improved the contiguity of both genome assemblies.

### Polishing, consensus and scaffolding

Genome assemblies were polished using the programs Pilon^69^ and Arrow (https://github.com/PacificBiosciences/GenomicConsensus). Polishing the contigs using both programs brings the error rate down to 0.01% or lower^66^. First, raw Illumina sequences were aligned to its corresponding merged assembly (draft genome) with Bowtie2^70^. We used SAMtools v1.8^71^ to transform, sort and index the alignments outputs and then Pilon was used to polishing the draft genome with these Illumina aligned reads.

We used the program pbalign (https://github.com/PacificBiosciences/pbalign) to align the PacBio raw sequences to the new corresponding polished draft genome from Pilon. Then, the program Arrow was implemented as a second polishing step and to generate a consensus genome. After this, we used the program OPERA-LG v2.0.6^72^ for scaffolding and then a third step of polishing with Pilon (which implied align the raw Illumina sequences against its corresponding genome) to improve the accuracy of the final genome assembly.

To evaluate the sequence and structural similarity between the two draft genomes (i.e., single nucleotide polymorphisms or SNPs, breakpoints, insertions, relocations, translocations, inversions, average sequence similarity), we used the wrapper dnadiff from MUMmer v3^24^. The Ticumán assembly was used as reference and the Teotihuacán assembly as query. Likewise, NOVOPlasty v3.8.2 was used to extract and reconstruct the chloroplast and mitochondrial genomes from the whole genome shotgun data of the two plants of *D. stramonium*. This program is capable to assemble the incidentally sequenced chloroplast and mitochondrial DNA that is present in almost all plant sequencing projects, due to the extraction of whole cellular DNA. The complete report and results of the chloroplast and mitochondrial genomes of these plants can be consulted in De-la-Cruz and Núñez-Farfán^73^.

### Nuclear genome validation

We evaluated the genome assemblies using the standard assembly statistics (average contig size, number of contigs, assembled genome size, N50, etc.) with the package Quast v5.0.2^74^. Also, BUSCO v.2.0.1^25^ was used to assess the assembly quality through the gene completeness for both genomes. BUSCO was run in its three assessment modes; genome, transcriptome and proteins. BUSCO inspects de novo assemblies searching for single-copy orthologs (BUSCOs) and assess the completeness of the genomes according with the number of BUSCOs found^25^. In our case, the “Solanaceae odb10*” dataset loaded in the program was used to find 3052 orthologs. BUSCOs were classified as complete and single-copy (S), complete and duplicated (D), fragmented (F) or missing (M). As an additional evaluation of the genome assembly quality, we assessed the mapping rate of the Illumina sequences of each individual to their corresponding assembly using Bowtie2. We also used the program Merqury v1.1^23^ to evaluate the quality of genome assemblies. Merqury generates assembly assessment metrics using k-mers alone^23^. This program compares a set of k-mers derived from unassembled, high accuracy sequencing reads to a genome assembly for evaluation (e.g., Illumina short-reads). The generated assembly metrics include consensus quality (QV) and k-mer completeness, Thus, Merqury is able to estimate base-level accuracy and completeness of genome assemblies^23^.

### Repetitive elements analysis

To characterize the repetitive elements in the genomes of *D. stramonium,* we followed the pipeline “Repeat Library Construction-Basic for MAKER v2.31.10” (http://weatherby.genetics.utah.edu/MAKER/wiki/index.php/Repeat_Library_Construction—Basic) by Campbell et al.^75^. With this method, we followed a de novo approach to identified and collect repetitive sequences from the genomes. This was achieved using RepeatModeler v2.0^76^. The repetitive elements derived from this pipeline were concatenated with the databases RepBase-20181026 (https://www.girinst.org/server/RepBase/index.php) and Dfam_Consensus-20181026 (https://dfam.org/help/tools). These databases contain a comprehensive number of repetitive elements from all plant species^76^. Then, the program RepeatMasker v4.0.9^77^ was run to identify the final interspersed repeats and low complexity DNA sequences. The output of the program was a detailed annotation of the repeats that are present in the genome sequence, as well as a modified version of the genome sequence in which all the annotated repeats have been masked^77^.

### Gene prediction and structural annotation

The program BUSCO was used for genome assembly assessment and the annotated BUSCO gene models built during genome assessments were used to optimize the Hidden Markov search model (HMM) to train the gene predictor program Augustus v.3.2.2^78^ using the—long option (BUSCO uses Augustus to search the conserved genes) and produce a trained HMM of our genes models for the program MAKER v.2.31.10^75^. MAKER identifies and masks out repeat elements based on repeat annotation from RepeatMasker, aligns RNA-seq data from the same species and/or related species to the genome; also, aligns proteins from related species and use gene predictors to synthesizes all these data into final structural annotations and produces evidence-based quality values for downstream annotation management^75^.

The *D. stramonium* annotation workflow consisted of a total of four MAKER runs which is the recommended number to obtain the best annotation^75^. For the first run, we used the gene trained models from Augustus, 443,235 proteins from UniProtKB/TrEMBL from all Solanaceae species database (searching for the word “Solanaceae” with date 30/03/2019), expression sequence tags (ESTs) and a transcriptome of *D. stramonium* provided by an alternative experiment from our laboratory (other plants; NCBI BioProject PRJNA669339), 328,166 ESTs of five Solanaceae species (*Solanum lycopersicum*, *Solanum tuberosum*, *Nicotiana attenuata*, *Nicotiana tabacum* and *Capsicum annuum*) from EnsemblPlants and our specific repeat library to masks out the genome. This first step produced a set of draft gene models. The gene models from this first run were used to train another ab initio gene predictor called SNAP v.2006-07-28^79^. Once SNAP was trained with the draft gene models, we ran MAKER again using the same parameters. This process was repeated twice to retrain SNAP for three times in total^79^. Therefore, we used the gene models from the one round to train ab initio SNAP program to improve the inference of gene models in the next round. Only the HMM gene models from SNAP in the MAKER configuration file was changed in each run. Retraining of SNAP was performed using gene models (from each previous MAKER run) with an annotation edit distance (AED ≤ 0.25) and amino acids length ≥ 50 bp. AED ranges from 0 to 1 and quantifies the congruency between a gene annotation and its supporting evidence (gene models, EST, protein and mRNAseq alignments)^80,81^. Lower AED values imply higher congruency between the intron–exon coordinates of annotation and its aligned evidence, whereas AED = 1 indicates no evidence for support of predicted genes^80^. Only sequences with AED < 0.5 were retained in the final set of predicted genes^80^. AED scores were calculated following the formulate given by Holt and Yandell^80^. We used the Perl scripts from the GitHub repository Genome Assembly Annotation Service (GAAS) (https://github.com/NBISweden/GAAS/tree/master/annotation) in order to retrieve AED scores and summary statistics from the MAKER annotation.

### Functional annotation

Blastp v.2.6.0^82^ was used to functionally annotate the genes from both *Datura* genomes against all the Solanaceae sequences from the UniProt/TrEMBL and UniProt/Swiss-Prot database. We used the program Automated Assignment of Human Readable Descriptions (AHRD) (https://github.com/asishallab/AHRD) to assign gene descriptions that were concise, informative and precise^10^. Gene Ontology terms were annotated using MapMan4 through the Mercator webtool^83^. In addition, protein motifs and domains were annotated using Interproscan v.5.24^84^, by searching against publicly available databases, including TIGRFAM^85^, SFLD^86^, ProDom^87^, CDD^88^, PRINTS^89^, PHANTER^90^, Gene3D^91^, PIRSF^92^, Coils^93^, MobiDB-lite^94^, PROSITE^95^, SMART^96^, SUPERFAMILY^97^, and Pfam^98^.

### Data sources for comparative genomics (gene family analysis)

Gene family analyses included 11 genomes representing almost all the Solanaceae species that have complete genomes as well as the two genomes of *D. stramonium*. Retrieval of protein coding genes and CDS from 11 genomes were sourced from the Sol Genomics Network (https://solgenomics.net/; *Nicotiana tabacum*^15^, *Nicotiana sylvestris*^11^, *Nicotiana attenuata*^26^, *Nicotiana tomentosiformis*^11^, *Solanum pimpinellifolium*^16^, *Solanum lycopersicum*^10^, *Solanum pennellii*^13^, *Solanum tuberosum*^9^, *Capsicum annuum*, CM334 v1.55^99^, *Capsicum annuum* var. *glabriusculum*^12^ and *Petunia inflata*^14^.

### Orthology, reconstruction of orthogroups (protein families) and construction of species and gene family trees

To gain insight into the evolution of *D. stramonium* genome, we used the thirteen proteomes as input to OrthoFinder program^29^. We used in OrthoFinder v2.3.3, DIAMOND blast (E-value < 1e^−5^)^100^ for orthogroup inference, and the MCL clustering algorithm for sequence similarity and clustering^101^. For each orthogroup or gene family we used MAFFT v7^102^ as multiple protein sequence aligner and FastTree2 v2.1.10^103^ for maximum likelihood gene trees inference. The inference of species tree is constructed by OrthoFinder, using a concatenated alignment of single-copy orthogroups (those with at most one gene per species)^29^. For some species sets which have been diverging for a very long time, there are not enough single copy orthogroups. In those cases, orthogroups that are mostly single-copy are also used for the concatenated alignments by only using sequences for the species that are single-copy in that orthogroup and gap characters for the other species^29^. The species tree was inferred with FastTree2^29^. The rooting is done via STRIDE algorithm (Specie Tree Root Inference from Duplication Events)^29^ and according with OrthoFinder, *P. inflata*, was selected as outgroup of the whole phylogeny.

### Inferring the species ultrametric phylogeny

To build an ultrametric phylogeny for the analysis of gene family evolution (expansions/contractions in gene families; see below), the rooted species tree obtained from OrthoFinder was used to search in TimeTree webtool^104^ the divergence times between the branches, the rooted species tree and the information of divergence times were used to create the ultrametric species tree using the chronos function of the R package ape (v.3.4 on R v.3.2.1)^105^. The tip to root length was adjusted to match the approximately 40 million-year evolutionary history of Solanaceae species^14,104^.

### Identification and analysis of gene expansions/contractions

To assess the gene family expansion and contractions of the thirteen Solanaceae species, we used only the gene families with more than four genes per family (24,235) and the species ultrametric tree as inputs to the CAFE v4.2.1^106^ open access program (Computational Analysis of gene Family Evolution). The main goal of CAFE is to estimate the birth–death (λ) parameters for the provided tree and gene family counts, the λ parameter describes the probability that any gene will be gained or lost^106^. First, the python scripts provided by CAFE pipeline were used to estimate the error in our dataset and to removed gene families with large variance^106^. This last filter was carried out because gene families that have large gene copy number variance can cause parameter estimates to be non-informative^106^. The CAFE software was then run using the mode in which the gain and loss rates are estimated together (λ) for the whole phylogeny. For the entire analysis, the CAFE overall *p* value threshold was kept at its default value (0.01). We used a custom script (https://github.com/asishallab/SlydGeneFamsAnalyses/blob/icruz/exec/parseCafeResult.R) to parse the CAFE output for functional enrichment analysis (see below).

### Physicochemical protein divergence

We used all the multiple sequence alignments of the 24,235 (protein families with more than four proteins) protein families to carried out a Multivariate Analysis of Protein Polymorphism (MAPP program)^107^. MAPP estimates the average deviation from six physicochemical properties (hydropathy, polarity, charge, volume, free energy in alpha-helix conformation, and free energy in beta-strand conformation) at an amino acid position across a multiple sequence alignment to assess the effect of a substitution at a particular amino acid site (physicochemical divergence)^107^. Thus, we used MAPP to estimate the physiochemical divergence in each gene family. First, we used the script readAndParseOrthogroupsTxt.R (https://github.com/asishallab/SlydGeneFamsAnalyses/blob/icruz/exec/readAndParseOrthogroupsTxt.R) to parse and create folders from each gene family and stored its corresponding protein tree and multiple sequence alignment from OrthoFinder results. Then, we used MAPP program^107^ with default parameters in each one of the protein families. We used the script readMappResults.R (https://github.com/asishallab/SlydGeneFamsAnalyses/blob/icruz/exec/readMappResults.R) to parse and read all the MAPP results of the gene families. This script reads the MAPP results for all families, adjust *p *value, find *Datura* genes of families with good multiple sequence alignments (Valdar Score > 0.6) and only retains significant sites with physicochemical divergence that fell into conserved domain proteins. Valdar Score method allows to score residues in a multiple sequence alignment and assigns a score ranging from 0 for low and 1 for high conservation^108^. This program can be found in https://github.com/asishallab/SlydGeneFamsAnalyses/blob/icruz/exec/computeValdarMsaScores. R and was used into the readMappResults.R script.

### Positive selection in gene families

We performed a codon-level analysis of positive natural selection with FUBAR program (Fast, Unconstrained Bayesian AppRoximation)^109^ on 24,235 gene families. FUBAR is a Bayesian approach to infer non-synoymous (dN) and synonymous (dS) substitution rates on a per-site basis for a given coding alignment and corresponding gene phylogeny^109^. To run FUBAR, first we retrieved the coding sequences (CDS) for each of the 13 Solanaceae species mentioned above. We removed trailing stop codons from the CDS, then we applied PAL2NAL^110^ to produce a codon alignment for each gene family. PAL2NAL is a program that converts a multiple sequence alignment of proteins and the corresponding DNA (CDS) sequences into a codon alignment^110^. Thus, we used the protein tree that we already had from each protein family to run PAL2NAL. FUBAR was run for all the codon alignments of each protein family. A custom Python script was used to transform the “.json” format from FUBAR result to tabular format. Then, the R script “loadFubarResults.R” from the R package GeneFamilies (https://github.com/asishallab/GeneFamilies/blob/master/exec/loadFubarResults.R) was used to obtain a table with the significant posterior probabilities of a codon being subject to positive selection for each gene family (significant posterior probabilities ≥ 0.98; and Bayes Factor > 100).

### Enrichment analysis

For enrichment test (Fisher's exact test^111^), we used as background all the proteins from both genomes of *D. stramonium* (64,790 proteins) to detect over-represented proteins that showed signal of expansion, physicochemical divergence (MAPP), and with positively selected conserved amino acids (codons) (FUBAR). Functional annotation of the proteins was done using MapMan4^83^ and InterproScan. MapMan4 was used to annotate the general function of the proteins in order to retrieve the function of significant proteins resulted from MAPP, FUBAR and CAFE analyses, while InterProscan was used to identify and annotate domains overlapping the proteins with significant expansion signal, proteins with physicochemical divergence as well as positively selected conserved amino acids (codons). These analyses were done using the scripts “enrichedAnnosInExpContrFams.R (CAFE)”, “identifyDomainsAtSelectedSites.R (FUBAR)” and “readMappResults.R (MAPP)” of the R package SlydGeneFamsAnalyses (https://github.com/asishallab/SlydGeneFamsAnalyses).

### Genes involved in the tropane alkaloids biosynthesis

We investigated four families that contain genes involved in the pathway of tropane alkaloids that is stored in the KEEG database; https://www.genome.jp/kegg-bin/show_pathway?map=map00960&show_description=show^22^). These genes correspond to Putrescine *N*-methyltransferase (*pmt*), Tropinone reductase I (*tpr* I), Tropinone reductase II (*tpr* II), and Hyoscyamine (6S)-dioxygenase (*h6h*). Multiple sequence alignments and protein trees for each family were generated from the previous analyses. We analyzed into our CAFE results if these eight protein families experienced expansions. Since proteins were already functional annotated, we also investigated the differences in the protein domain architecture in each gene family.

It is important to note that the gene family storing *h6h* contained just two genes belonging to both *D. stramonium* genomes. Since special interest was pointed out to the gene *h6h*, we retrieved 13 *h6h* genes from UniProt database belong to *Datura metel* (acc. Q6EZB3), *D. stramonium* Acc A0A0M4K1P1 (acc. A0A0M4K1P1), *Brugmansia arborea* (acc. A0A0M3SG09), *Hyoscyamus niger* (acc. P24397), *Brugmansia x candida* (hybrid plant generated by *Brugmansia aurea* x *Brugmansia versicolor*, acc. B2CNC8), *Hyoscyamus senecionis* (J7HDC2), *Atropa baetica* (acc. A9Q1G4), *Atropa belladonna* (acc. *Q9XJ43*), *Capsicum chinense* (acc. A0A2G3CG79), *Medicago truncatula* (acc. I3SNT9), *Glycine soja* (acc. A0A0B2P514), *Vitis vinifera* (acc. A0A438KDU2) and *Zea mays* (acc. B6T4W5). These genes were joined as a *h6h* gene family for which we generated a multiple sequence alignment with MAFT and a gene tree using FastTree2 with default parameters. Domain architecture was annotated with Pfam 31.0 database.

## Supplementary Information

Supplementary Information.

## Data Availability

The complete workflow, all supplemental materials as well as commands used in this study are available in https://github.com/icruz1989/Datura-stramonium-genome-project. Genome assemblies, Illumina and PacBio raw sequences from the two plants of *D. stramonium* have been deposited at DDBJ/ENA/GenBank under the BioProject PRJNA622882: Teotihuacan assembly, *acc*. JAAWWX000000000, Ticumán assembly *acc*. JAAWWY000000000. Illumina and PacBio sequences for the Ticumán genome: *acc*. SRR11474700, SRR11474698, respectively. Illumina and PacBio sequences for the Teotihuacán genome: SRR11474701, SRR11474699, respectively.

## References

[1] Chowański SZ (2016). A review of bioinsecticidal activity of Solanaceae alkaloids. Toxins.

[2] Kohnen-Johannsen KL, Kayser O (2019). Tropane alkaloids: chemistry, pharmacology, biosynthesis and Production. Molecules.

[3] De-la-Cruz IM (2020). Evolutionary response to herbivory: population differentiation in microsatellite loci, tropane alkaloids and leaf trichome density in *Datura stramonium*. Arthropod-Plant Interact..

[4] Hightower CE (1979). Plants that kill and cure. Vet. Hum. Toxicol..

[5] WHO (2015). Annex 1 19th WHO Model List of Essential Medicines.

[6] Barclay, A. S. Studies in the genus *Datura* (Solanaceae) I. Taxonomy of subgenus *Datura*. Ph.D. Thesis. Harvard University, Cambridge, MA, USA (1959).

[7] Symon, D. E. & Haegi, L. *Datura* (Solanaceae) is a New world genus (eds. Hawkes, J. G., Lester, R. N., Nee, M. & Estrada, R. N.) 197–210 (The Royal Botanic Gardens, 1991).

[8] Weaver SE, Warwick SI (1984). The biology of Canadian weeds: 64 *Datura stramonium* L. Can. J. Plant Sci.

[9] Xu X (2011). Genome sequence and analysis of the tuber crop potato. Nature.

[10] Sato S (2012). The tomato genome sequence provides insights into fleshy fruit evolution. Nature.

[11] Sierro N (2013). Reference genomes and transcriptomes of *Nicotiana sylvestris* and *Nicotiana tomentosiformis*. Genome Biol..

[12] Qin C (2014). Whole-genome sequencing of cultivated and wild peppers provides insights into *Capsicum* domestication and specialization. Proc. Natl. Acad. Sci..

[13] Bolger A (2014). The genome of the stress-tolerant wild tomato species *Solanum pennellii*. Nat. Genet..

[14] Bombarely A (2016). Insight into the evolution of the Solanaceae from the parental genomes of *Petunia hybrida*. Nat. Plants.

[15] Edwards KD (2017). A reference genome for *Nicotiana tabacum* enables map-based cloning of homologous loci implicated in nitrogen utilization efficiency. BMC Genomics.

[16] Razali R (2018). The genome sequence of the wild tomato *Solanum pimpinellifolium* provides insights into salinity tolerance. Front. Plant Sci..

[17] Barchi L (2019). A chromosome-anchored eggplant genome sequence reveals key events in Solanaceae evolution. Sci. Rep..

[18] Savolainen O, Lascoux M, Merilä J (2013). Ecological genomics of local adaptation. Nat. Rev. Genet..

[19] Valverde PL, Fornoni J, Núñez-Farfán J (2003). Evolutionary ecology of *Datura stramonium*: equal plant fitness benefits of growth and resistance against herbivory. J. Evol. Biol..

[20] Fornoni J, Valverde PL, Nunez-Farfan J (2004). Population variation in the cost and benefit of tolerance and resistance against herbivory in *Datura stramonium*. Evolution.

[21] De-la-Cruz IM (2020). Genomic and chemical evidence for local adaptation in resistance to different herbivores in *Datura stramonium*. Evolution.

[22] Kanehisa M, Sato Y (2019). KEGG Mapper for inferring cellular functions from protein sequences. Protein Sci..

[23] Rhie A (2020). Merqury: reference-free quality, completeness, and phasing assessment for genome assemblies. BioRxiv.

[24] Kurtz S (2004). Versatile and open software for comparing large genomes. Genome Biol..

[25] Simão FA (2015). BUSCO: assessing genome assembly and annotation completeness with single-copy orthologs. Bioinformatics.

[26] Xu S (2017). Wild tobacco genomes reveal the evolution of nicotine biosynthesis. Proc. Natl. Acad. Sci..

[27] Hulse-Kemp AM (2018). Reference quality assembly of the 3.5-Gb genome of *Capsicum annuum* from a single linked-read library. Hortic. Res..

[28] Kubis S, Schmidt T, Heslop-Harrison JS (1998). Repetitive DNA Elements as a major component of plant genomes. Ann. Bot..

[29] Emms DM, Kelly S (2019). OrthoFinder: phylogenetic orthology inference for comparative genomics. Genome Biol..

[30] Särkinen T (2013). A phylogenetic framework for evolutionary study of the nightshades (Solanaceae): a dated 1000-tip tree. BMC Evol. Biol..

[31] Rairdan GJ, Moffett P (2006). Distinct domains in the ARC region of the potato resistance protein Rx mediate LRR binding and inhibition of activation. Plant Cell.

[32] Tameling WIL, Baulcombe DC (2007). Physical association of the NB-LRR resistance protein Rx with a ran GTPase Activating protein is required for extreme resistance to Potato virus. Plant Cell.

[33] van Ooijen G (2008). Structure–function analysis of the NB-ARC domain of plant disease resistance proteins. J. Exp. Bot..

[34] Padmanabhan M, Cournoyer P, Dinesh-Kumar SP (2009). The leucine-rich repeat domain in plant innate immunity: a wealth of possibilities. Cell. Microbiol..

[35] Magalhães DM (2016). LRR-RLK family from two Citrus species: genome-wide identification and evolutionary aspects. BMC Genomics.

[36] Afzal AJ, Wood A, Lightfoot DA (2008). Plant receptor-like serine threonine kinases: roles in signaling and plant defense. Mol. Plant Microbe Interact..

[37] Stone JM, Walker JC (1995). Plant protein kinase families and signal transduction. Plant Physiol..

[38] Ballvora A (2002). The R1 gene for potato resistance to late blight (*Phytophthora infestans*) belongs to the leucine zipper/NBS/LRR class of plant resistance genes. Plant J..

[39] Bischoff V (2010). Trichome Birefringence and its homolog AT5G01360 encode plant-specific DUF231 proteins required for cellulose biosynthesis in *Arabidopsis*. Plant Physiol..

[40] Liu Q (2019). The cytochrome P450 CYP72A552 is key to production of hederagenin-based saponins that mediate plant defense against herbivores. New Phytol..

[41] Degenhardt J (2003). Attracting friends to feast on foes: engineering terpene emission to make crop plants more attractive to herbivore enemies. Curr. Opin. Biotechnol..

[42] Tholl D (2006). Terpene synthases and the regulation, diversity and biological roles of terpene metabolism. Curr. Opin. Plant. Biol..

[43] Cheng AX (2007). Plant terpenoids: biosynthesis and ecological functions. J. Integr. Plant Biol..

[44] Mithöfer A, Boland W (2012). Plant defense against herbivores: chemical Aspects. Annu. Rev. Plant Biol..

[45] Heil M, Karban R (2010). Explaining evolution of plant communication by airborne signals. Trends Ecol. Evol..

[46] Shanker AK (2014). Drought stress responses in crops. Funct. Integr. Genomics.

[47] Thangella PAV (2018). Differential expression of leaf proteins in four cultivars of peanut (*Arachis hypogaea* L.) under water stress. 3 Biotechnology.

[48] Kawahara Y (2017). Galactose oxidase/kelch repeat-containing protein is involved in the iron deficiency stress response in the roots of *Hyoscyamus albus*. Plant Root.

[49] Li R (2006). Functional genomic analysis of alkaloid biosynthesis in *Hyoscyamus niger* reveals a cytochrome P450 involved in littorine rearrangement. Chem. Biol..

[50] Nasomjai P (2009). Mechanistic insights into the Cytochrome P450-mediated oxidation and rearrangement of littorine in tropane alkaloid biosynthesis. ChemBioChem.

[51] Kasukabe Y (2004). Overexpression of spermidine synthase enhances tolerance to multiple environmental stresses and up-regulates the expression of various stress-regulated genes in transgenic *Arabidopsis thaliana*. Plant Cell Physiol..

[52] Wei Z (2000). Sulfakinins reduce food intake in the desert locust *Schistocerca gregaria*. J. Insect Physiol..

[53] Massengo-Tiassé RP, Cronan JE (2009). Diversity in enoyl-acyl carrier protein reductases. Cell. Mol. Life Sci..

[54] Shidore T, Triplett LR (2017). Toxin–antitoxin systems: implications for plant disease. Annu. Rev. Phytopathol..

[55] Ramisetty BCM, Santhosh RS (2016). Horizontal gene transfer of chromosomal Type II toxin–antitoxin systems of *Escherichia coli*. FEMS Microbiol. Lett..

[56] Roth J, Lawrence J, Bobik T (1996). Cobalamin (Coenzyme B12): synthesis and biological significance. Annu. Rev. Microbiol..

[57] Qin L (2020). Molecular cloning and functional analysis of hyoscyamine 6β-hydroxylase (H6H) in the poisonous and medicinal plant *Datura innoxia* mill. Plant. Physiol. Biochem..

[58] Shonle I, Bergelson J (2000). Evolutionary ecology of the tropane alkaloids of *Datura stramonium* (Solanaceae). Evolution.

[59] Castillo G (2014). Selection mosaic exerted by specialist and generalist herbivores on chemical and physical defense of *Datura stramonium*. PLoS ONE.

[60] Miranda-Pérez A (2016). Natural selection drives chemical resistance of *Datura stramonium*. PeerJ.

[61] Doyle JJ, Doyle JL (1987). A rapid DNA isolation procedure for small quantities of fresh leaf tissue. Phytochem. Bull..

[62] Salzberg SL (2012). GAGE: a critical evaluation of genome assemblies and assembly algorithms. Genome Res..

[63] Bolger AM, Lohse M, Usadel B (2014). Trimmomatic: a flexible trimmer for Illumina sequence data. Bioinformatics.

[64] Andrews, S. FastQC: a quality control tool for high throughput sequence data. http://www.bioinformatics.babraham.ac.uk/projects/fastqc (2010). Accessed November 2018.

[65] Chikhi R, Medvedev P (2013). Informed and automated k-mer size selection for genome assembly. Bioinformatics.

[66] Chakraborty M (2016). Contiguous and accurate *de novo* assembly of metazoan genomes with modest long read coverage. Nucleic Acids Res..

[67] Koren S (2017). Canu: scalable and accurate long-read assembly via adaptive k-mer weighting and repeat separation. Genome Res..

[68] Ye C (2016). DBG2OLC: Efficient assembly of large genomes using long erroneous reads of the third-generation sequencing technologies. Sci. Rep..

[69] Walker BJ (2014). Pilon: an integrated tool for comprehensive microbial variant detection and genome assembly improvement. PLoS ONE.

[70] Langmead B (2018). Scaling read aligners to hundreds of threads on general-purpose processors. Bioinformatics.

[71] Li H (2009). The sequence alignment/map format and SAMtools. Bioinformatics.

[72] Gao S (2016). OPERA-LG: efficient and exact scaffolding of large, repeat-rich eukaryotic genomes with performance guarantees. Genome Biol..

[73] De-la-Cruz IM, Núñéz-Farfán J (2020). The complete chloroplast genomes of two Mexican plants of the medicinal and toxic herb *Datura Stramonium* (Solanaceae). Mitochondrial DNA Part B.

[74] Gurevich A (2013). QUAST: quality assessment tool for genome assemblies. Bioinformatics.

[75] Campbell MS (2014). Genome annotation and curation using MAKER and MAKER-P. Curr. Protocol Bioinform..

[76] Smit, A. F. A. & Hubley, R. RepeatModeler Open-1.0. http://www.repeatmasker.org (2008–2015). Accessed April 2019.

[77] Smit, A. F. A., Hubley, R. & Green, P. RepeatMasker Open-4.0. http://www.repeatmasker.org (2013–2015). Accessed May 2019.

[78] Stanke M (2006). AUGUSTUS: ab initio prediction of alternative transcripts. Nucleic Acids Res..

[79] Korf I (2004). Gene finding in novel genomes. BMC Bioinform..

[80] Holt C, Yandell M (2011). MAKER2: An annotation pipeline and genome-database management tool for second-generation genome projects. BMC Bioinform..

[81] Ozerov MY (2018). Highly continuous genome assembly of Eurasian Perch (*Perca fluviatilis*) using linked-read sequencing. G3.

[82] Boratyn GM (2013). BLAST: a more efficient report with usability improvements. Nucleic Acids Res..

[83] Schwacke R (2019). MapMan4: a refined protein classification and annotation framework applicable to multi-omics data analysis. Mol. Plant.

[84] Jones P (2014). InterProScan 5: genome-scale protein function classification. Bioinformatics.

[85] Haft DH (2013). TIGRFAMs and genome properties in 2013. Nucleic Acids Res..

[86] Akiva ES (2014). The structure-function linkage database. Nucleic Acids. Res..

[87] Bru C (2005). The ProDom database of protein domain families: more emphasis on 3D. Nucleic Acids Res..

[88] Marchler-Bauer A (2017). CDD/SPARCLE: functional classification of proteins via subfamily domain architectures. Nucleic Acids Res..

[89] Attwood TK (2012). The PRINTS database: a fine-grained protein sequence annotation and analysis resource, its status in 2012. Database.

[90] Thomas PD (2003). PANTHER: a library of protein families and subfamilies indexed by function. Genome Res..

[91] Yeats C (2006). Gene3D: modelling protein structure, function and evolution. Nucleic Acids Res..

[92] Nikolskaya AN (2007). PIRSF family classification system for protein functional and evolutionary analysis. Evol. Bioinform..

[93] Lupas AV, Van Dyke M, Stock J (1991). Predicting coiled coils from protein sequences. Science.

[94] Necci M (2017). MobiDB-lite: fast and highly specific consensus prediction of intrinsic disorder in proteins. Bioinformatics.

[95] Sigrist CJA (2013). New and continuing developments at PROSITE. Nucleic Acids Res..

[96] Letunic I, Doerks T, Bork P (2012). SMART 7: recent updates to the protein domain annotation resource. Nucleic Acids Res..

[97] de Lima Morais DA (2011). SUPERFAMILY 1.75 including a domain-centric gene ontology method. Nucleic Acids Res..

[98] Finn RD (2014). Pfam: the protein families database. Nucleic Acids Res..

[99] Kim S (2014). Genome sequence of the hot pepper provides insights into the evolution of pungency in *Capsicum* species. Nat. Genet..

[100] Buchfink B, Xie C, Huson D (2015). Fast and sensitive protein alignment using DIAMOND. Nat. Methods.

[101] Enright A, Dongen S, Ouzounis CA (2002). An efficient algorithm for large-scale detection of protein families. Nucleic Acids Res..

[102] Katoh M, Kuma M (2002). MAFFT: a novel method for rapid multiple sequence alignment based on fast Fourier transform. Nucleic Acids. Res..

[103] Price MN, Dehal PS, Arkin AP (2010). FastTree 2 approximately maximum-likelihood trees for large alignments. PLoS ONE.

[104] Kumar S (2017). TimeTree: a resource for timelines, timetrees, and divergence times. Mol. Biol. Evol..

[105] Paradis E, Schliep K (2019). ape 5.0: An environment for modern phylogenetics and evolutionary analyses in R. Bioinformatics.

[106] Han MV (2013). Estimating gene gain and loss rates in the presence of error in genome assembly and annotation using CAFE 3. Mol. Biol. Evol..

[107] Stone EA, Sidow A (2005). Physicochemical constraint violation by missense substitutions mediates impairment of protein function and disease severity. Genome Res..

[108] Valdar WS (2002). Scoring residue conservation. Proteins.

[109] Murrell B (2013). FUBAR: a fast, unconstrained Bayesian approximation for inferring selection. Mol. Biol. Evol..

[110] Suyama M, Torrents D, Bork P (2006). PAL2NAL: robust conversion of protein sequence alignments into the corresponding codon alignments. Nucleic Acids Res..

[111] Fisher RA (1922). On the interpretation of χ^2^ from contingency tables, and the calculation of *P*. J. R. Stat. Soc..

