# Supplementary material for: Genomic signatures of the evolution of defence against its natural enemies in the poisonous and medicinal plant Datura stramonium (Solanaceae)
Source: Sci Rep. 2021 Jan 13;11:882. doi: 10.1038/s41598-020-79194-1 (PMC7806989; doi:10.1038/s41598-020-79194-1)

Supplementary information file of the manuscript "Genomic signatures of the evolution of defence against its natural enemies in the poisonous and medicinal plant *Datura stramonium* (Solanaceae)"

I. M. De-la-Cruz, A. Hallab, U. Olivares-Pinto, R. Tapia-López, S. Velázquez-Márquez, D. Piñero, K. Oyama, B. Usadel, J. Núñez-Farfán

Scientific Reports

Supplementary Table S1. DNA reads produced for *Datura stramonium* genome assembly

Library technology	Plant ID	Sequences	Number of total sequences	Length of the sequences (bp)	Trimmed sequences	Length of the trimmed sequences	% GC	Coverage
Illumina HiSeq 4000	Teotihuacán	PE reads	159,031,241	150	151,898,993	36-151	39	~ 30.29 X
	Ticumán	PE reads	161,985,268	150	152,665,899	36-151	41	~ 30.85 X
PacBio Sequel	Teotihuacán	SMRTbell library Subreads	9,505,413	~ 9,000-15,000	--	--	39	~ 20 X
	Ticumán		9,995,713	~ 9,000-18,000	--	--	39	~ 20 X

Supplementary Table S2. Flow cytometry values of two *Datura stramonium* plants.

**Sample ID**	**VC G1 *Datura stramonium***	**VC G1 PBMCs**	**C-value Datura (2C) Mbp**
Ticumán 23	2.04	2.03	1,709 – 1,994
Ticumán 23 (2)	2.03	2.25	1,724 – 2,011
Ticumán 23 (3)	2.71	2.30	1,720 – 2,007
Teotihuacán 1	2.63	2.57	1,720 – 2,007
Teotihuacán 1 (1)	2.67	2.87	1,744 – 2,035
Teotihuacán 2 (2)	3.26	2.52	1,730 – 2,018

Supplementary Table S3. Single-copy othologs (BUSCOs) searched in (a) genome, (b) proteome, and (c) transcriptome mode for both Genomes of *Datura stramonium*.

BUSCO statistics	Ticumán	Teotihuacán
**(a) Genome-wide**		
Complete BUSCOs (c)	2,779	2,493
Complete and single-copy BUSCOs (S)	2,693	2,418
Complete and duplicated BUSCOs (D)	86	75
Fragmented BUSCOs (F)	128	189
Missing BUSCOs (M)	145	370
Total BUSCO groups searched	3,052	3,052
**(b) Proteome**		
Complete BUSCOs (c)	2,703	2,398
Complete and single-copy BUSCOs (S)	2,601	2,320
Complete and duplicated BUSCOs (D)	102	78
Fragmented BUSCOs (F)	194	301
Missing BUSCOs (M)	155	353
Total BUSCO groups searched	3,052	3,052
**(c) Transcriptome**		
Complete BUSCOs (c)	2,735	2,439
Complete and single-copy BUSCOs (S)	2,639	2,367
Complete and duplicated BUSCOs (D)	96	72
Fragmented BUSCOs (F)	180	275
Missing BUSCOs (M)	137	338
Total BUSCO groups searched	3,052	3,052

Supplementary Fig. S1. Merqury copy number spectrum (spectra-cn) of the same k-mers in (a), colored by copy numbers found in the Teotihuacán assembly and (b) Ticumán assembly. (c) Distinct k-mer assembly spectrum (spectra-asm) plot of both Teotihuacán and Ticumán assemblies. This plot shows the unique (blue and green) and shared portion of k-mers (purple).

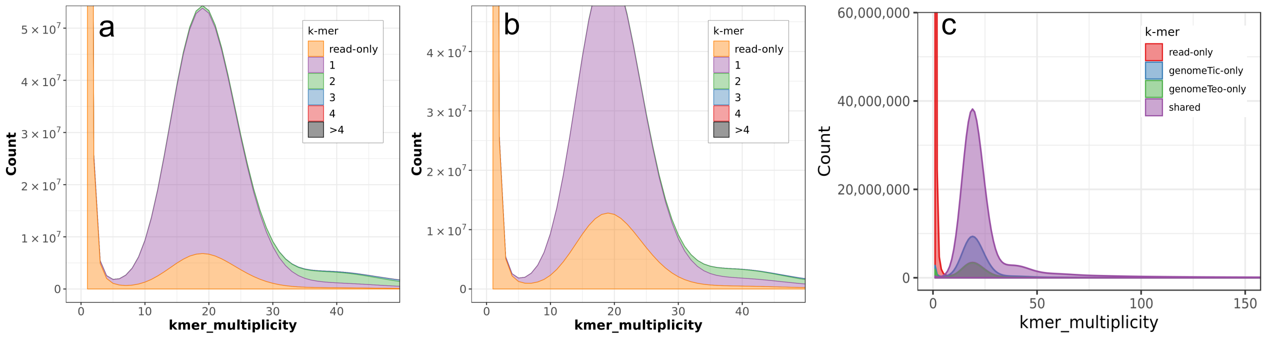

Supplementary Table S4. Overall alignment rate of raw short paired-end sequences to its corresponding assembly of both *Datura stramonium* plants.

Sequences	Ticumán		Teotihuacán	
	#	%	#	%
Total	152,665,899	100	151,898,993	100
Aligned concordantly 0 times	37,997,103	24.89	42,519,632	27.99
Aligned concordantly exactly 1 time	55,789,233	36.54	56,107,428	36.94
Aligned concordantly >1 times	58,879,563	38.57	53,271,933	35.07
Pairs aligned concordantly 0 times	37,997,103	-	42,519,632	-
Aligned discordantly 1 time	8,023,876	21.12	6,437,203	15.14
pairs aligned 0 times concordantly or discordantly	29,973,227	-	36,082,429	-
mates make up the pairs	59,946,454	-	72,164,858	-
aligned 0 times	11,785,149	19.66	31,995,085	44.34
aligned exactly 1 time	8,332,932	13.90	9,833,367	13.63
aligned >1 times	39,828,373	66.44	30,336,406	42.4
overall alignment rate	-	96.14	-	89.47

Supplementary Table S5. Alignment between the two *Datura stramonium* genomes. REF = reference, QRY = Query.

**dnadiff analysis**	**[REF] TICUMÁN GENOME**	**[QRY] TEOTIHUACÁN GENOME**
Sequences		
TotalSeqs	27915	30392
AlignedSeqs	26062(93.36%)	29871(98.29%)
UnalignedSeqs	1853(6.64%)	521(1.71%)
Bases		
TotalBases	1482573840	1288887151
AlignedBases	1292570395(87.18%)	1141549386(88.57%)
UnalignedBases	190003445(12.82%)	147337765(11.43%)
Alignments		
1-to-1	89169	89169
TotalLength	1065922758	1070788774
AvgLength	11953.96	12008.53
AvgIdentity	97.92	97.92
M-to-M	342597	342597
TotalLength	1510387351	1516161954
AvgLength	4408.64	4425.5
AvgIdentity	95.69	95.69
Feature	Estimates	
Breakpoints	676003	674038
Relocations	2232	1297
Translocations	16246	10044
Inversions	150	146
Insertions	336122	193938
InsertionSum	510172288	260588913
InsertionAvg	1517.82	1343.67
TandemIns	352	259
TandemInsSum	188427	115414
TandemInsAvg	535.3	445.61
SNPs		
TotalSNPs	6673981	6673981
GT	757129(11.34%)	959438(14.38%)
GC	182368(2.73%)	180196(2.70%)
GA	501238(7.51%)	555611(8.33%)
TG	959438(14.38%)	757129(11.34%)
TA	393108(5.89%)	388352(5.82%)
TC	555094(8.32%)	502444(7.53%)
CA	756874(11.34%)	942129(14.12%)
CG	180196(2.70%)	182368(2.73%)
CT	502444(7.53%)	555094(8.32%)
AG	555611(8.33%)	501238(7.51%)
AT	388352(5.82%)	393108(5.89%)
AC	942129(14.12%)	756874(11.34%)
TotalGSNPs	770516	770516
TG	44215(5.74%)	39180(5.08%)
TA	31182(4.05%)	31542(4.09%)
TC	131117(17.02%)	120495(15.64%)
GA	120663(15.66%)	131634(17.08%)
GC	18623(2.42%)	18709(2.43%)
GT	39180(5.08%)	44215(5.74%)
CA	39019(5.06%)	44137(5.73%)
CG	18709(2.43%)	18623(2.42%)
CT	120495(15.64%)	131117(17.02%)
AC	44137(5.73%)	39019(5.06%)
AG	131634(17.08%)	120663(15.66%)
AT	31542(4.09%)	31182(4.05%)
TotalIndels	14347656	14347656
G.	855437(5.96%)	2030087(14.15%)
T.	1526304(10.64%)	2744958(19.13%)
C.	870348(6.07%)	2017384(14.06%)
A.	1541685(10.75%)	2760998(19.24%)
N.	208(0.00%)	247(0.00%)
.A	2760998(19.24%)	1541685(10.75%)
.C	2017384(14.06%)	870348(6.07%)
.N	247(0.00%)	208(0.00%)
.G	2030087(14.15%)	855437(5.96%)
.T	2744958(19.13%)	1526304(10.64%)
TotalGIndels	478792	478792
T.	72370(15.12%)	115642(24.15%)
G.	25948(5.42%)	25051(5.23%)
N.	3(0.00%)	43(0.01%)
C.	25408(5.31%)	25126(5.25%)
A.	74361(15.53%)	114840(23.99%)
.N	43(0.01%)	3(0.00%)
.C	25126(5.25%)	25408(5.31%)
.A	114840(23.99%)	74361(15.53%)
.G	25051(5.23%)	25948(5.42%)
.T	115642(24.15%)	72370(15.12%)

Supplementary Table S6. Number of elements, length occupied and percentage of sequence of the repeat elements in the genome assemblies of both *Datura stramonium* individuals identified by RepeatModeler and RepeatMasker.

Repeat class	Number of elements	Length occupied (bp)	Percentage of sequence	Number of elements	Length occupied (bp)	Percentage of sequence
	Ticumán				Teotihuacán	
SINEs	16,542	4,402,707	0.30	18,140	3,331,482	0.26
LINEs:	43,108	23,439,474	1.58	46,737	23,593,403	1.83
LTR	534,845	976,671,332	65.88	492,830	817,336,298	63.41
DNA elements	162,833	48,087,803	3.24	171,198	46,018,189	3.57
Unclassified	114,168	52,221,075	3.52	115,278	46,571,233	3.61
Small RNA	14,535	5,997,859	0.40	16,014	6,557,612	0.51
Satellites	2,934	875,492	0.06	2,956	661,284	0.05
Simple repeats	184,608	12,181,935	0.82	171,120	10,393,978	0.81
Low complexity	43,493	3,524,503	0.24	44,144	3,089,946	0.24
Total	1,117,066	1,127,402,180	76.04	1,078,417	957,553,425	74.11

Supplementary Table S7. Repetitive elements for 10 Solanaceae species and their proportion in the genomes as well as the proportion in the genome of long terminal repeat (LTR) elements.

Species	Repeats	Genome size	LTR retroelements and retrotransposons
	% assembled genome	Mb	%
Solanum lycopersicum	68	900	61
Solanum tuberosum	62.20	844	54.35
Petunia axilaris	63.08	1380	40.41
Perunia inflata	59.22	1430	36.98
Nicotiana tomentosiformis	74.84	2360	52.21
Nicotiana sylvestris	71.95	2680	48.65
Nicotiana attenuata	81	2370	81
Capsicum annum	76.36	3480	58.11
Datura stramonium acc. Ticumán	76.04	1470	74.52
Datura stramonium acc. Teotihuacán	74.11	1280	58.71

Supplementary Table S8. OrthoFinder statistics of the gene families construction using 13 Solanaceae plant proteomes.

OrthoFinder statistics	#
Number of genes	536483
Number of genes in orthogroups	480594
Number of unassigned genes	55889
Percentage of genes in orthogroups	89.6
Percentage of unassigned genes	10.4
Number of orthogroups	35458
Number of species-specific orthogroups	540
Number of genes in species-specific orthogroups	2186
Percentage of genes in species-specific orthogroups	0.4
Mean orthogroup size	13.6
Median orthogroup size	11.0
G50 (assigned genes)	21
G50 (all genes)	19
O50 (assigned genes)	6653
O50 (all genes)	8077
Number of orthogroups with all species present	10141
Number of single-copy orthogroups	181

Supplementary Table S9. Full list of InterproScan domains with signal of physicochemical divergence in the genome of *Datura stramonium*.

Domain	*p*-value adjusted	Annotation name
IPR025771	4.28E-07	Phosphoethanolamine N-methyltransferase
IPR001944	4.18E-04	Glycoside hydrolase, family 35
IPR031330	3.64E-04	Glycoside hydrolase 35, catalytic domain
IPR008979	8.52E-03	Galactose-binding-like domain superfamily
IPR012562	1.14E-06	GUCT
IPR035979	1.35E-04	RNA-binding domain superfamily
IPR016040	1.76E-05	NAD(P)-binding domain
IPR008166	2.38E-03	Glycosyltransferase family 92
IPR006011	1.01E-03	Syntaxin, N-terminal domain
IPR000727	7.30E-03	Target SNARE coiled-coil homology domain
IPR006012	1.04E-03	Syntaxin/epimorphin, conserved site
IPR009291	9.74E-05	Vacuolar protein sorting-associated protein 62
IPR030070	4.74E-06	SNF1-related protein kinase regulatory subunit beta-2
IPR006828	2.36E-05	Association with the SNF1 complex (ASC) domain
IPR037256	2.36E-05	ASC domain superfamily
IPR026147	4.77E-03	Rab3 GTPase-activating protein catalytic subunit
IPR001494	5.59E-04	Importin-beta, N-terminal domain
IPR003689	8.15E-04	Zinc/iron permease
IPR004698	6.00E-05	Zinc/iron permease, fungal/plant
IPR002346	1.64E-08	Molybdopterin dehydrogenase, FAD-binding
IPR016166	1.85E-04	FAD-binding domain, PCMH-type
IPR036318	3.54E-04	FAD-binding, type PCMH-like superfamily
IPR006058	2.98E-04	2Fe-2S ferredoxin, iron-sulphur binding site
IPR036010	5.30E-06	2Fe-2S ferredoxin-like superfamily
IPR005107	1.64E-08	CO dehydrogenase flavoprotein, C-terminal
IPR036683	1.64E-08	CO dehydrogenase flavoprotein, C-terminal domain superfamily
IPR000674	1.64E-08	Aldehyde oxidase/xanthine dehydrogenase, a/b hammerhead
IPR036856	1.64E-08	Aldehyde oxidase/xanthine dehydrogenase, a/b hammerhead superfamily
IPR039877	1.04E-03	Transmembrane protein 131-like
IPR002659	7.03E-03	Glycosyl transferase, family 31
IPR025298	5.29E-03	Domain of unknown function DUF4094
IPR001900	7.03E-03	Ribonuclease II/R
IPR023208	4.77E-03	NADPH-cytochrome P450 reductase
IPR019987	4.77E-03	GTP-binding protein, ribosome biogenesis, YsxC
IPR030393	7.03E-03	EngB-type guanine nucleotide-binding (G) domain
IPR001533	4.77E-03	Pterin 4 alpha carbinolamine dehydratase
IPR036428	4.77E-03	Pterin 4 alpha carbinolamine dehydratase superfamily
IPR031099	9.44E-03	BRCA1-associated
IPR034016	1.04E-03	Aminopeptidase N-type
IPR024571	1.04E-03	ERAP1-like C-terminal domain
IPR004125	9.44E-03	Signal recognition particle, SRP54 subunit, M-domain
IPR036891	9.44E-03	Signal recognition particle, SRP54 subunit, M-domain superfamily
IPR006195	2.09E-03	Aminoacyl-tRNA synthetase, class II
IPR022228	7.03E-03	Protein of unknown function DUF3755
IPR029981	4.77E-03	Trichome birefringence-like 45/PMR5
IPR013783	2.08E-03	Immunoglobulin-like fold
IPR022100	1.04E-03	Minichromosome loss protein Mcl1, middle region
IPR016208	1.64E-08	Aldehyde oxidase/xanthine dehydrogenase
IPR008274	1.64E-08	Aldehyde oxidase/xanthine dehydrogenase, molybdopterin binding

Supplementary Table S10. Full list of InterproScan domains with signal of expansion in the genome of *Datura stramonium*.

Domains	*p*-value adjusted	Annotation name
IPR032675	1.17E-05	Leucine-rich repeat domain superfamily
IPR036396	7.89E-03	Cytochrome P450 superfamily
IPR002401	4.54E-03	Cytochrome P450, E-class, group I
IPR001128	5.18E-03	Cytochrome P450
IPR008543	1.14E-25	Uncharacterised protein family Ycf2
IPR019557	8.03E-11	Aminotransferase-like, plant mobile domain
IPR006904	3.54E-06	Protein of unknown function DUF716
IPR036397	3.47E-03	Ribonuclease H superfamily
IPR015300	2.28E-03	DNA-binding pseudobarrel domain superfamily
IPR003480	8.57E-05	Transferase
IPR023213	5.06E-04	Chloramphenicol acetyltransferase-like domain superfamily
IPR008949	1.91E-04	Isoprenoid synthase domain superfamily
IPR036408	3.88E-04	Photosystem I PsaA/PsaB superfamily
IPR001280	1.19E-05	Photosystem I PsaA/PsaB
IPR007125	5.79E-03	Histone H2A/H2B/H3
IPR009072	5.20E-07	Histone-fold
IPR001810	1.05E-07	F-box domain
IPR036047	1.11E-09	F-box-like domain superfamily
IPR017451	1.03E-21	F-box associated interaction domain
IPR036879	1.21E-35	Transcription factor, MADS-box superfamily
IPR002100	3.39E-35	Transcription factor, MADS-box
IPR012337	1.13E-04	Ribonuclease H-like superfamily
IPR012340	4.15E-17	Nucleic acid-binding, OB-fold
IPR036691	9.74E-10	Endonuclease/exonuclease/phosphatase superfamily
IPR001951	7.06E-03	Histone H4
IPR019809	2.27E-03	Histone H4, conserved site
IPR000194	2.31E-04	ATPase, F1/V1/A1 complex, alpha/beta subunit, nucleotide-binding domain
IPR036121	8.27E-03	ATPase, F1/V1/A1 complex, alpha/beta subunit, N-terminal domain superfamily
IPR023366	2.70E-04	ATP synthase subunit alpha, N-terminal domain-like superfamily
IPR038376	5.22E-04	ATP synthase, alpha subunit, C-terminal domain superfamily
IPR000793	7.65E-04	ATP synthase, alpha subunit, C-terminal
IPR001906	1.70E-05	Terpene synthase, N-terminal domain
IPR036965	1.36E-04	Terpene synthase, N-terminal domain superfamily
IPR008930	1.14E-03	Terpenoid cyclases/protein prenyltransferase alpha-alpha toroid
IPR005630	5.06E-05	Terpene synthase, metal-binding domain
IPR025558	1.80E-11	Domain of unknown function DUF4283
IPR038005	2.85E-13	Virus X resistance protein-like, coiled-coil domain
IPR041118	3.41E-04	Rx, N-terminal
IPR002182	9.74E-10	NB-ARC
IPR006936	1.95E-03	ALOG domain
IPR040222	5.41E-03	ALOG family
IPR004000	7.16E-09	Actin family
IPR004001	9.95E-06	Actin, conserved site
IPR020902	2.98E-11	Actin/actin-like conserved site
IPR002156	2.86E-11	Ribonuclease H domain
IPR006243	3.07E-05	Photosystem I PsaA
IPR034741	8.81E-03	Terpene cyclase-like 1, C-terminal domain
IPR007641	4.13E-04	RNA polymerase Rpb2, domain 7
IPR015712	3.29E-14	DNA-directed RNA polymerase, subunit 2
IPR037033	2.25E-07	DNA-directed RNA polymerase, subunit 2, hybrid-binding domain superfamily
IPR007120	1.68E-07	DNA-directed RNA polymerase, subunit 2, hybrid-binding domain
IPR008896	1.71E-21	Protein TIC214
IPR006534	7.06E-03	P-type ATPase, subfamily IIIA
IPR006527	1.08E-05	F-box associated domain, type 1
IPR023393	3.88E-04	START-like domain superfamily
IPR000916	4.28E-08	Bet v I/Major latex protein
IPR003008	1.00E-04	Tubulin/FtsZ, GTPase domain

IPR000217	2.18E-06	Tubulin
IPR036525	1.16E-03	Tubulin/FtsZ, GTPase domain superfamily
IPR011043	9.96E-03	Galactose oxidase/kelch, beta-propeller
IPR020586	8.81E-03	Photosystem I PsaA/PsaB, conserved site
IPR037034	3.84E-03	RNA polymerase Rpb2, domain 2 superfamily
IPR007642	4.13E-04	RNA polymerase Rpb2, domain 2
IPR007645	1.11E-03	RNA polymerase Rpb2, domain 3
IPR000477	7.65E-04	Reverse transcriptase domain
IPR040256	1.82E-11	Uncharacterized protein At4g02000-like
IPR014724	5.22E-04	RNA polymerase Rpb2, OB-fold
IPR000569	8.19E-07	HECT domain
IPR035983	8.19E-07	HECT, E3 ligase catalytic domain
IPR013838	3.53E-04	Beta tubulin, autoregulation binding site
IPR006564	4.40E-04	Zinc finger, PMZ-type
IPR018289	6.38E-10	MULE transposase domain
IPR027806	2.60E-03	Harbinger transposase-derived nuclease domain
IPR004252	2.63E-20	Probable transposase, Ptta/En/Spm, plant
IPR021929	5.06E-05	Late blight resistance protein R1
IPR003918	7.06E-03	NADH:ubiquinone oxidoreductase
IPR029014	1.57E-04	[NiFe]-hydrogenase, large subunit
IPR038290	1.57E-04	NADH-quinone oxidoreductase, subunit D superfamily
IPR001135	8.57E-05	NADH-quinone oxidoreductase, subunit D
IPR022997	8.81E-03	NADH-quinone oxidoreductase chain 4
IPR008906	3.96E-05	HAT, C-terminal dimerisation domain
IPR013955	2.97E-09	Replication factor A, C-terminal
IPR003569	1.55E-03	Probable cytochrome c biosynthesis protein, plants
IPR004332	6.33E-13	Transposase, MuDR, plant
IPR029480	3.53E-04	Transposase-associated domain
IPR040339	3.53E-04	Uncharacterized membrane protein At1g16860-like
IPR026960	1.95E-18	Reverse transcriptase zinc-binding domain
IPR034583	2.27E-03	Protein EMBRYONIC FLOWER 1
IPR033251	3.53E-04	Protein NUCLEAR FUSION DEFECTIVE 6, chloroplastic/mitochondrial
IPR009632	8.55E-03	NA
IPR003871	9.63E-15	Domain of unknown function DUF223
IPR013921	8.55E-03	Mediator complex, subunit Med20
IPR031147	7.06E-03	Double-stranded RNA-binding protein
IPR007216	8.81E-03	CCR4-NOT transcription complex subunit 9

Supplementary Table S11. Full list of InterproScan domains with signal of positive selection in the genome of *Datura stramonium*.

Domains	Pp-value adjusted	Annotation name
IPR032675	4.32E-18	Leucine-rich repeat domain superfamily
IPR003653	2.29E-07	Ulp1 protease family, C-terminal catalytic domain
IPR036396	2.13E-10	Cytochrome P450 superfamily
IPR002401	4.05E-12	Cytochrome P450, E-class, group I
IPR001128	1.03E-10	Cytochrome P450
IPR017972	1.41E-08	Cytochrome P450, conserved site
IPR015410	7.01E-13	Domain of unknown function DUF1985
IPR010987	3.43E-07	Glutathione S-transferase, C-terminal-like
IPR040079	4.54E-04	Glutathione Transferase family
IPR004045	9.96E-07	Glutathione S-transferase, N-terminal
IPR004046	1.97E-05	Glutathione S-transferase, C-terminal
IPR036282	1.11E-05	Glutathione S-transferase, C-terminal domain superfamily
IPR036397	3.97E-04	Ribonuclease H superfamily
IPR003480	6.31E-04	Transferase
IPR023213	3.27E-03	Chloramphenicol acetyltransferase-like domain superfamily
IPR008949	1.65E-05	Isoprenoid synthase domain superfamily
IPR001810	1.16E-07	F-box domain
IPR036047	2.09E-12	F-box-like domain superfamily
IPR017451	1.79E-28	F-box associated interaction domain
IPR036879	4.24E-25	Transcription factor, MADS-box superfamily
IPR002100	4.24E-25	Transcription factor, MADS-box
IPR012337	1.00E-04	Ribonuclease H-like superfamily
IPR012340	8.43E-21	Nucleic acid-binding, OB-fold
IPR036691	1.68E-09	Endonuclease/exonuclease/phosphatase superfamily
IPR000194	6.85E-05	ATPase, F1/V1/A1 complex, alpha/beta subunit, nucleotide-binding domain
IPR038376	4.86E-05	ATP synthase, alpha subunit, C-terminal domain superfamily
IPR000793	7.71E-05	ATP synthase, alpha subunit, C-terminal
IPR001906	2.30E-07	Terpene synthase, N-terminal domain
IPR036965	3.43E-07	Terpene synthase, N-terminal domain superfamily
IPR008930	2.16E-07	Terpenoid cyclases/protein prenyltransferase alpha-alpha toroid
IPR005630	2.29E-06	Terpene synthase, metal-binding domain
IPR025558	1.56E-34	Domain of unknown function DUF4283
IPR038005	1.12E-17	Virus X resistance protein-like, coiled-coil domain
IPR041118	4.93E-09	Rx, N-terminal
IPR002182	5.18E-21	NB-ARC
IPR004001	6.62E-07	Actin, conserved site
IPR020902	4.34E-04	Actin/actin-like conserved site
IPR002156	1.07E-07	Ribonuclease H domain
IPR034741	2.53E-06	Terpene cyclase-like 1, C-terminal domain
IPR006534	2.57E-03	P-type ATPase, subfamily IIIA
IPR006527	1.11E-05	F-box associated domain, type 1
IPR000916	9.02E-04	Bet v I/Major latex protein
IPR011043	2.34E-03	Galactose oxidase/kelch, beta-propeller
IPR040256	1.54E-30	Uncharacterized protein At4g02000-like
IPR023409	1.85E-05	14-3-3 protein, conserved site
IPR036815	6.84E-05	14-3-3 domain superfamily
IPR023410	6.84E-05	14-3-3 domain
IPR000308	6.84E-05	14-3-3 protein
IPR004252	7.01E-13	Probable transposase, Ptta/En/Spm, plant
IPR023329	6.64E-03	Chlorophyll a/b binding domain superfamily
IPR001344	6.64E-03	Chlorophyll A-B binding protein, plant
IPR013955	9.74E-14	Replication factor A, C-terminal
IPR004332	1.25E-04	Transposase, MuDR, plant
IPR026960	4.86E-05	Reverse transcriptase zinc-binding domain
IPR003871	2.97E-14	Domain of unknown function DUF223
IPR013921	2.37E-03	Mediator complex, subunit Med20

Supplementary Table S12. Expanded gene families in the genome of *Datura stramonium* (annotation with MapMan 4).

BINCODE	NAME
2.4.4.2.5.2	Cellular respiration.oxidative phosphorylation.cytochrome c.CCM cytochrome c maturation system (system I).apocytochrome-heme assembly complex.CcmFn component
9.1.3.1	Secondary metabolism.terpenoids.terpenoid synthesis.mono-/sesquiterpene-/diterpene synthase
12.1.3	Chromatin organisation.histones.H2B-type histone
12.1.4	Chromatin organisation.histones.H3-type histone
12.1.5	Chromatin organisation.histones.H4-type histone
12.3.5.1.3	Chromatin organisation.histone modifications.histone ubiquitination.PRC1 histone mono-ubiquitination complex.Psc-type EMF1 component
15.3.6.1.7	RNA biosynthesis.RNA polymerase II-dependent transcription.MEDIATOR transcription co-activator complex.head module.MED20 component
15.5.5	RNA biosynthesis.siRNA biogenesis.CLSY3/4 regulator protein
15.7.14	RNA biosynthesis.transcriptional activation.MADS box transcription factor
20.2.1	Cytoskeleton.microfilament network.actin filament protein
24.1.1.1.2	Solute transport.primary active transport.V-type ATPase complex.membrane V0 subcomplex.subunit c
24.1.2.3.1	Solute transport.primary active transport.P-type ATPase superfamily.P3 family.AHA P3A-type proton-translocating ATPase
35.2	not assigned.not annotated
50.2.3	Enzyme classification.EC_2 transferases.EC_2.3 acyltransferase

Supplementary Table S13. Positively selected gene families in the genome of *Datura stramonium* (annotation with MapMan 4).

BINCODE	NAME
1.1.1.1.1	Photosynthesis.photophosphorylation.photosystem II.LHC-II complex.LHCb1/2/3-type component
5.1.9.2	Lipid metabolism.fatty acid synthesis.fatty acid desaturation and elongation.omega-3/omega-6 fatty acid desaturase
7.3.1	Coenzyme metabolism.S-adenosyl methionine (SAM) cycle.S-adenosyl methionine synthetase
9.1.3.1	Secondary metabolism.terpenoids.terpenoid synthesis.mono-/sesquiterpene-/diterpene synthase
11.4.3.1	Phytohormones.cytokinin.conjugation and degradation.UDP-dependent glycosyl transferase
12.1.3	Chromatin organisation.histones.H2B-type histone
15.3.6.1.7	RNA biosynthesis.RNA polymerase II-dependent transcription.MEDIATOR transcription co-activator complex.head module.MED20 component
15.7.14	RNA biosynthesis.transcriptional activation.MADS box transcription factor
18.8.1.12	Protein modification.phosphorylation.TKL kinase superfamily.LRR-XII kinase
18.8.1.24.1	Protein modification.phosphorylation.TKL kinase superfamily.G-Lectin kinase families.SD-1 kinase
18.12.1.4	Protein modification.S-glutathionylation and deglutathionylation.glutathione S-transferase activities.class tau
19.4.1.5.4.<br>1.4.6	Protein degradation.peptide tagging.Ubiquitin (UBQ)-anchor addition (ubiquitylation).UBQ-ligase E3 activities.Cullin-based ubiquitylation complexes.SKP1-CUL1-FBX (SCF) E3 ligase complexes.F-BOX substrate adaptor components.FBX component
19.4.5.2	Protein degradation.peptide tagging.Membrane-anchored-Ubiquitin (MUB)-anchor addition.UBC-subclass-6 conjugation E2 protein
20.2.1	Cytoskeleton.microfilament network.actin filament protein
24.1.2.3.1	Solute transport.primary active transport.P-type ATPase superfamily.P3 family.AHA P3A-type proton-translocating ATPase
24.1.4.1	Solute transport.primary active transport.VHP PPase family.VHP1 proton-translocating pyrophosphatase
24.2.10.2	Solute transport.carrier-mediated transport.OPT family.oligopeptide transporter (OPT-type)
26.3.2.3.1	External stimuli response.temperature.Hsp (heat-shock-responsive protein) families.Hsp70 family.DnaK protein
26.3.2.5.2	External stimuli response.temperature.Hsp (heat-shock-responsive protein) families.sHsp (small heat-shock-responsive protein) families.class-C-II protein
35.2	not assigned.not annotated
50.1.1	Enzyme classification.EC_1 oxidoreductases.EC_1.1 oxidoreductase acting on CH-OH group of donor
50.1.13	Enzyme classification.EC_1 oxidoreductases.EC_1.14 oxidoreductase acting on paired donor with incorporation or reduction of molecular oxygen
50.2.3	Enzyme classification.EC_2 transferases.EC_2.3 acyltransferase

Supplementary Table S14. Gene families with physicochemical divergence in the genome of *Datura stramonium* (annotation with MapMan. 4).

BINCODE	NAME
1.1.1.2.9	Photosynthesis.photophosphorylation.photosystem II.PS-II complex.component PsbR
1.1.1.6.2.2	Photosynthesis.photophosphorylation.photosystem II.LHC-related protein groups.two-helix LHC-related protein group.SEP2 protein
1.2.1.2.6	Photosynthesis.calvin cycle.ribulose-1,5-bisphosphat carboxylase/oxygenase (RuBisCo) activity.RuBisCo assembly.RAF2 assembly factor
2.4.1.4.6	Cellular respiration.oxidative phosphorylation.NADH dehydrogenase complex.non-core components.carbonic anhydrase component
3.1.2.7	Carbohydrate metabolism.sucrose metabolism.synthesis.sucrose-phosphate synthase
3.8.5.1	Carbohydrate metabolism.nucleotide sugar biosynthesis.UDP-D-xylose synthesis.UDP-D-glucuronic acid decarboxylase
3.8.8	Carbohydrate metabolism.nucleotide sugar biosynthesis.UDP-D-glucose 4-epimerase
5.2.3.2.1	Lipid metabolism.glycerolipid synthesis.phosphatidylcholine.methylation pathway.phosphatidylethanolamine N-methyltransferase
6.1.2.4	Nucleotide metabolism.purines.catabolism.xanthine dehydrogenase
7.8.3.1	Coenzyme metabolism.prenylquinone synthesis.plastoquinone synthesis.SPS3 solanesyl diphosphate synthase
9.3.3.2.5	Secondary metabolism.nitrogen-containing secondary compounds.glucosinolates.glucosinolate degradation.nitrilase
10.1.2	Redox homeostasis.reactive oxygen generation.xanthine dehydrogenase
11.3.1.1	Phytohormones.brassinosteroid.synthesis.steroid 22-alpha-hydroxylase (DWF4)
11.6.3.1	Phytohormones.gibberellin.conjugation and degradation.gibberellin modification enzyme
12.5.4.2.2	Chromatin organisation.DNA methylation.ROS1-mediated DNA demethylation.MBD7 ROS1-recruitment complex.IDM1 component
14.2.1	DNA damage response.BRCA1,ÄìBARD1 DNA-damage response heterodimer.BRCA1|BARD1 component
15.3.2.7.1.1	RNA biosynthesis.RNA polymerase II-dependent transcription.pre-initiation complex.TATA box-binding protein (TBP) regulation.NC2 regulator complex.alpha component
15.5.2	RNA biosynthesis.siRNA biogenesis.RDR2 ssRNA polymerase
15.7.4.7.1	RNA biosynthesis.transcriptional activation.bZIP superfamily.TGA transcription factor activity.TGA transcription factor
15.7.16	RNA biosynthesis.transcriptional activation.C3H zinc finger transcription factor
15.8.2	RNA biosynthesis.transcriptional repression.LUG transcriptional co-repressor
16.4.5.2.1	RNA processing.RNA splicing.spliceosome-associated non-snRNP MOS4-associated complex (MAC).associated components.MAC5 component
16.4.6.3	RNA processing.RNA splicing.spliceosome-associated non-snRNP factors.RS31/40/41 splicing factor
16.5.1.2.2	RNA processing.ribonuclease activities.RNase P ribonuclease activities.RNA-dependent RNase P complex.RPP30/POP2 component
16.10.2.2	RNA processing.organelle machineries.ribonuclease activities.RNase R exoribonuclease
17.2.4	Protein biosynthesis.aminoacyl-tRNA synthetase activities.aspartate-tRNA ligase
17.2.17	Protein biosynthesis.aminoacyl-tRNA synthetase activities.proline-tRNA ligase
18.8.1.29.1	Protein modification.phosphorylation.TKL kinase superfamily.URK kinase families.URK-1 kinase
18.8.1.34	Protein modification.phosphorylation.TKL kinase superfamily.RLCK-IV kinase
18.8.2.1	Protein modification.phosphorylation.STE kinase superfamily.MAPKK kinase
18.8.5.1.2	Protein modification.phosphorylation.CAMK kinase superfamily.SNF1-related SnRK1 kinase complex.beta-type regulatory subunit
18.10.1.1.6	Protein modification.dephosphorylation.serine/threonine protein phosphatase superfamily.PPP Fe-Zn-dependent phosphatase families.PP7 phosphatase
18.10.1.2.4	Protein modification.dephosphorylation.serine/threonine protein phosphatase superfamily.PPM/PP2C Mn/Mg-dependent phosphatase families.clade D phosphatase
19.5.5.6.5	Protein degradation.peptidase families.metallopeptidase activities.aminopeptidase activities.M1 neutral/aromatic-hydroxyl amino acid aminopeptidase
20.4.3.1	Cytoskeleton.cytoskeleton-nucleoskeleton linking.nuclear lamina.CRWN lamin-like protein
21.3.2.1.2	Cell wall.pectin.rhamnogalacturonan I.synthesis.beta-1,4-galactosyltransferase
21.3.2.2.1	Cell wall.pectin.rhamnogalacturonan I.modification and degradation.beta-galactosidase
21.3.2.2.2.2	Cell wall.pectin.rhamnogalacturonan I.modification and degradation.alpha-L-arabinofuranosidase activities.bifunctional BXL-type alpha-L-arabinofuranosidase and beta-D-xylosidase
21.4.1.1.1.3	Cell wall.cell wall proteins.hydroxyproline-rich glycoproteins.arabinogalactan proteins (AGPs).glycosylation.AGP beta-1,3-galactosyltransferase
21.9.1.4	Cell wall.cutin and suberin.cuticular lipid formation.omega-hydroxy fatty acyl dehydrogenase
22.6.2.1	Vesicle trafficking.endomembrane trafficking.vacuolar sorting.VSR vacuolar sorting receptor
22.6.2.2.1	Vesicle trafficking.endomembrane trafficking.vacuolar sorting.AP-4 vacuole cargo adaptor complex.AP4E large epsilon subunit
22.8.1.1	Vesicle trafficking.SNARE target membrane recognition and fusion complexes.Qa-type SNARE components.SYP1-group protein
23.1.7.3	Protein translocation.chloroplast.thylakoid membrane SRP insertion system.cpSRP54 component
23.5.1.2.6	Protein translocation.nucleus.nucleocytoplasmic transport.karyopherin beta transport receptors.KA120 import karyopherin
23.5.1.2.14	Protein translocation.nucleus.nucleocytoplasmic transport.karyopherin beta transport receptors.TNPO3 transport karyopherin
24.1.2.2.2	Solute transport.primary active transport.P-type ATPase superfamily.P2 family.ACA P2B-type calcium cation-transporting ATPase
24.2.2.7	Solute transport.carrier-mediated transport.MFS superfamily.PHT4 phosphate transporter
24.2.7.1.2	Solute transport.carrier-mediated transport.IT superfamily.DASS family.dicarboxylate:malate antiporter (DIT-type)
24.2.11.1	Solute transport.carrier-mediated transport.ZIP family.metal cation transporter (ZIP-type)
25.1.6.1	Nutrient uptake.nitrogen assimilation.glutamate deamination.glutamate dehydrogenase
26.1.1.1	External stimuli response.light.red/far red light.PHY phytochrome photoreceptor
27.3.1.2	Multi-process regulation.SnRK1 metabolic regulator system.SnRK1 kinase complex.beta regulatory subunit
50.1.6	Enzyme classification.EC_1 oxidoreductases.EC_1.6 oxidoreductase acting on NADH or NADPH

Supplementary Table S15. Classifications of domains related with abiotic stresses subject to expansion, positive selection or physicochemical divergence. Some domains were detected to be expanded and positively selected. Ex = expanded, PS = positive selected, FQ = physicochemical divergence. *p*-value is showed for each analysis. The entire list for each analysis is showed in Supplementary Tables S9-S11.

Domains related with abiotic streesess	InterproScan	*p*-value	Analysis	Function
SNF1-related protein kinase regulatory subunit beta-2	IPR030070	4.740E-06	FQ	Drought, carbohydrate metabolism, glucose limitation, assimilation of nitrogen
Association with the SNF1 complex (ASC) domain	IPR006828	2.360E-05	FQ	Glucose limitation, nutritional stress
ASC domain superfamily	IPR037256	2.360E-05	FQ	Glucose limitation
Zinc/iron permease	IPR003689	8.146E-04	FQ	Symbiotic nitrogen fixation, acquisition, distribution, homeostasis, and signaling of Zn
Zinc/iron permease, fungal/plant	IPR003689	8.146E-04	FQ	Mycorrhizal interaction
Molybdopterin dehydrogenase, FAD-binding	IPR016166	1.854E-04	FQ	Purine degradation and stress response
2Fe-2S ferredoxin, iron-sulphur binding site	IPR006058	2.983E-04	FQ	Photosynthesis, biosynthesis of chlorophyll, phytochrome and fatty acids, assimilation of sulphur and nitrogen
2Fe-2S ferredoxin-like superfamily	IPR036010	5.300E-06	FQ	Photosynthesis, biosynthesis of chlorophyll, phytochrome and fatty acids, assimilation of sulphur and nitrogen
CO dehydrogenase flavoprotein, C-terminal	IPR005107	1.640E-08	FQ	Blue light, seedling establishment, phototropism and circadian clock regulation
CO dehydrogenase flavoprotein, C-terminal domain superfamily	IPR036683	1.640E-08	FQ	Blue light, seedling establishment, phototropism and circadian clock regulation
Aldehyde oxidase/xanthine dehydrogenase, molybdopterin binding	IPR008274	1.640E-08	FQ	Biosynthesis of abscisic acid and indole-3-acetic acid, freezing, drought and salinity, purine metabolism
NADH:ubiquinone oxidoreductase	IPR003918	7.056E-03	FQ	Reducing nitrate to nitrite
NADH-quinone oxidoreductase, subunit D superfamily	IPR038290	1.571E-04	FQ	Reducing nitrate to nitrite
NADH-quinone oxidoreductase, subunit D	IPR001135	8.570E-05	FQ	Reducing nitrate to nitrite
NADH-quinone oxidoreductase chain 4	IPR022997	8.806E-03	FQ	Reducing nitrate to nitrite
DNA-binding pseudobarrel domain superfamily	IPR015300	2.277E-03/1.646E-05	E, PS	Plant growth and development, modeling of root architecture, and development of seeds
Isoprenoid synthase domain superfamily	IPR008949	1.909E-04/1.646E-05	E, PS	Carbon Partitioning Within the Cytoplasmic Pathway
Photosystem I PsaA/PsaB superfamily	IPR036408	3.878E-04	E	Uses light energy to mediate electron transfer from plastocyanin to ferredoxin
Photosystem I PsaA/PsaB	IPR001280	1.190E-05	E	Uses light energy to mediate electron transfer from plastocyanin to ferredoxin
Photosystem I PsaA	IPR006243	3.070E-05	E	Uses light energy to mediate electron transfer from plastocyanin to ferredoxin
Photosystem I PsaA/PsaB, conserved site	IPR020586	8.806E-03	E	Uses light energy to mediate electron transfer from plastocyanin to ferredoxin
F-box domain	IPR001810	1.048E-07/1.155E-07	E, PS	Floral transition as well as panicle and seed development and floral transition as well as panicle and seed development
F-box-like domain superfamily	IPR036047	1.114E-09/2.094E-12	E, PS	Floral transition as well as panicle and seed development and floral transition as well as panicle and seed development
F-box associated interaction domain	IPR017451	1.032E-21/1.788E-28	E, PS	Floral transition as well as panicle and seed development and floral transition as well as panicle and seed development
Transcription factor, MADS-box superfamily	IPR036879	1.212E-35/4.235E-25	E, PS	Floral development
Transcription factor, MADS-box	IPR002100	3.387E-35/4.235E-25	E, PS	Floral development
F-box associated domain, type 1	IPR006527	1.075E-05/1.109E-05	E, PS	Floral development
ALOG domain	IPR006936	1.949E-03	E	Key developmental regulators
ALOG family	IPR040222	5.408E-03	E	Key developmental regulators
START-like domain superfamily	IPR023393	3.878E-04	E	Response to drought, salt, wound and heat stress, stress response
Galactose oxidase/kelch, beta-propeller	IPR011043	9.958E-03/2.340E-03	E, PS	Stress responses induced under Fe deficiency in the roots
HAT, C-terminal dimerisation domain	IPR008906	3.960E-05	E	Abiotic and biotic stress
Protein EMBRYONIC FLOWER 1	IPR034583	2.271E-03	E	Controls leaves development and shoot architecture by delaying both the vegetative to reproductive transition
Protein NUCLEAR FUSION DEFECTIVE 6	IPR033251	3.534E-04	E	Nuclear fusion in sexual reproduction
Double-stranded RNA-binding protein	IPR031147	7.056E-03	E	Plant hormone signaling
Ulp1 protease family, C-terminal catalytic domain	IPR003653	2.290E-07	E	Salt stress signaling
Domain of unknown function DUF4283	IPR025558	1.559E-34/2.967E-14	E, PS	Cell wall biology, vasculature patterning and abiotic/biotic stress response
Chlorophyll a/b binding domain superfamily	IPR023329	6.638E-03	PS	Light receptor that captures and delivers excitation energy to photosystems I and II
Chlorophyll A-B binding protein, plant	IPR001344	6.638E-03	PS	Light receptor that captures and delivers excitation energy to photosystems I and II
Reverse transcriptase zinc-binding domain	IPR026960	4.860E-05	PS	Leaf senescence

Supplementary Table S16. Expansions detected from CAFE analysis of four tropane alkaloid genes of *Datura stramonium*.

Gene: Putrescine N-methyltransferase (*pmt*)

Orthogroup: OG0002818

CAFE result

(Pi_3:35,(((Date_2:0.1,Dati_3:0.1)_1:30,((Cag_2:1.3,Cam_2:1.3)_2:17.7,(Stu_2:7.9,(Spe_2:3.6,(Sly_2:1.5,Spi_2:1.5)_2:2.1)_2:4.3)_2:11.1)_2:11)_1:1,(Nto_1:10,(Nat_2:7,(Nsy_4:4.2,Ntab_3:4.2)_3:2.7)_2:3)_2:21)_1:4)_1 0
((0.0535963,0.556162),(0,0),(0.765557,0.0707071),(0.529328,0.529328),(0.775253,0.716815),(0.659885,0.603046),(0.580611,0.556114),(0.529328,0.529328),(0.515,0.114623),(0.308472,0.580611),(0.659885,0.0297541),(0.086099,0.642348))

Gene: Tropinone reductase I (*tpr* I)

Orthogroup: OG0000734

cag13003 cag24876 cag8773 cag8774 cam17363 cam27165 cam27166 cam27167 date11128 date20542 date9161 date9170 dati22507 dati33027 dati33033 dati33044 natt24258 natt24289 natt32224 nsy24327 nsy41968 nsy41969 nsy41970 nta20644 nta37371 nta43555 nta60093 ntom17820 ntom17821 ntom999 pin17463 pin3196 pin33888 pin35337 sly10744 sly10747 sly20505 sly20507 spe14195 spe14202 spe27158 spe27159 spim10448 spim10450 spim19733 spim19735 stu18780 stu18782 stu18783 stu18784 stu8512

CAFE result

(Pi_4:35,(((Date_4:0.1,Dati_4:0.1)_4:30,((Cag_4:1.3,Cam_4:1.3)_4:17.7,(Stu_5:7.9,(Spe_4:3.6,(Sly_4:1.5,Spi_4:1.5)_4:2.1)_4:4.3)_4:11.1)_4:11)_4:1,(Nto_3:10,(Nat_3:7,(Nsy_4:4.2,Ntab_4:4.2)_4:2.7)_4:3)_4:21)_4:4)_4 0.992 ((-,-),(-,-),(-,-),(-,-),(-,-),(-,-),(-,-),(-,-),(-,-),(-,-),(-,-),(-,-))

Gene: Tropinone reductase II (*tpr* II)

Orthogroup: OG0000791

CAFE result

cag2058 cag2590 cag30171 cam11768 cam18109 cam20945 date10377 date10388 date10390 date27832 date754 dati10564 dati23798 dati23799 natt116 natt19071 natt21503 natt33056 natt8539 nsy36242 nta35121 nta38873 nta55266 nta55579 ntom28776 ntom35271 ntom35272 pin11074 pin11076 pin23774 sly26258 sly26259 sly30392 spe16039 spe34851 spe34852 spe34853 spe41475 spe41476 spim12021 spim25311 spim25312 spim29439 stu11416 stu26582 stu26583 stu26584 stu28553 stu28554

Gene: Hyoscyamine_6_dioxygenase (*h6h*)

CAFE result

Orthogroup: OG0028637

(Pi_0:35,(((Date_1:0.1,Dati_1:0.1)_1:30,((Cag_0:1.3,Cam_0:1.3)_0:17.7,(Stu_0:7.9,(Spe_0:3.6,(Sly_0:1.5,Spi_0:1.5)_0:2.1)_0:4.3)_0:11.1)_0:11)_1:1,(Nto_0:10,(Nat_0:7,(Nsy_0:4.2,Ntab_0:4.2)_0:2.7)_0:3)_0:21)_1:4)_1 0.88 ((-,-),(-,-),(-,-),(-,-),(-,-),(-,-),(-,-),(-,-),(-,-),(-,-),(-,-),(-,-))

Supplementary Fig. S2. Putrescine N-methyltransferase (*pmt*) gene tree. date = *Datura stramonium* Teotihuacán. dati = *Datura stramonium* Ticumán.

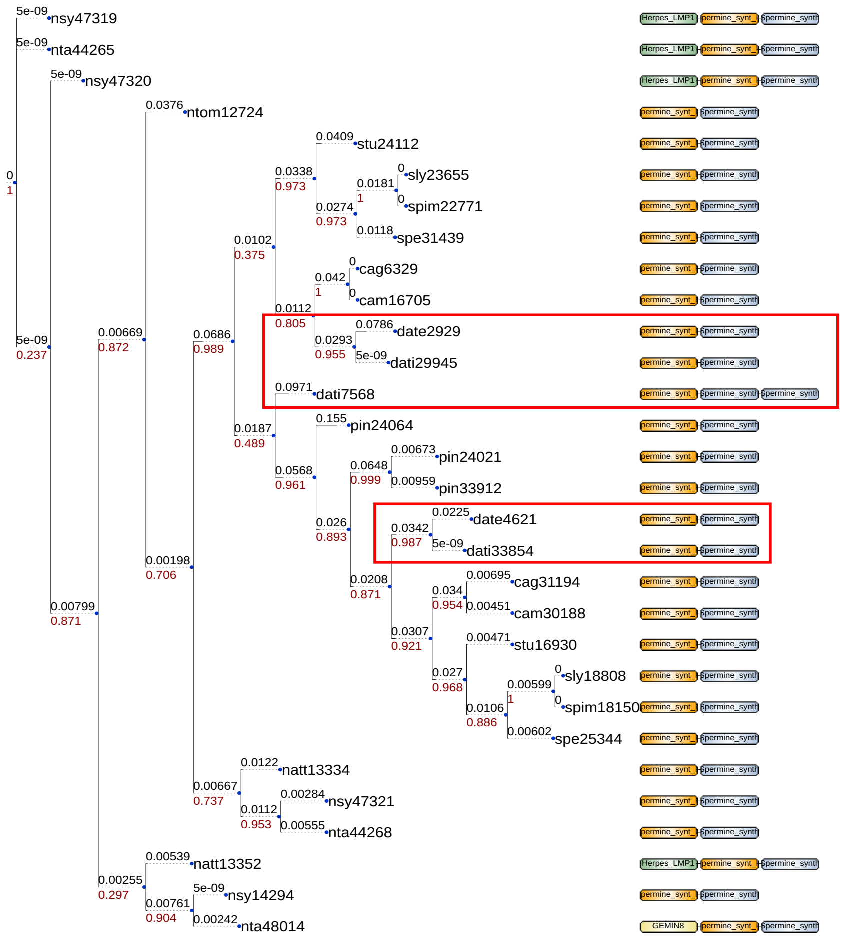

Supplementary Table S17. Chemical differentiation between both genomes of *Datura stramonium*.

Genome	Teotihuacán 1	Ticumán 23	Ratio
Atropine	230.61778	1010.87812	4.3833486
3-Phenylacetoxy-6,7-Epoxytropane	110.285784	26599.4799	241.186842
3-Tigloyloxy-6-hidroxytropane	0	1290.11976	0
3-Hidroxy- 6-Tigloyloxytropane	308.650108	0	0
Hygrine	5.84875045	155.798535	26.6379181
Tropine	5.3	169.362577	31.9552032
Scopoline	18.7091197	4079.14967	218.030016
Scopine	12.7840543	147.646212	11.5492479
Cyclotropine	38.092288	477.775824	12.5425867
Tropinone	118.446686	95.0197785	0.80221559
Atropine impurity E	18.4205943	2783.1221	151.087531
Anisodamine	80.9036681	11905.7377	147.15943
6,7-Dehydroyoscyamine	9.88993948	126.031923	12.7434474
6-Hydroxyapoatropine	6.90143575	98.9808131	14.3420611
Apoatropine	0	92.9162373	0
Aposcopolamine	0	203.020399	0
3-(3′-Methoxytropoyloxy) tropane	0	304.155792	0
3-Phenylacetoxytropane	9.22068556	136.041075	14.7539003
Scopolamine	44.2938921	9375.96887	211.676338
Total alkaloid concentration	1018.36478	59051.2053	57.9862994

Supplementary Fig. S3. Tropinone reductase II (*tpr* II) gene tree. date = *Datura stramonium* Teotihuacán. dati = *Datura stramonium* Ticumán.

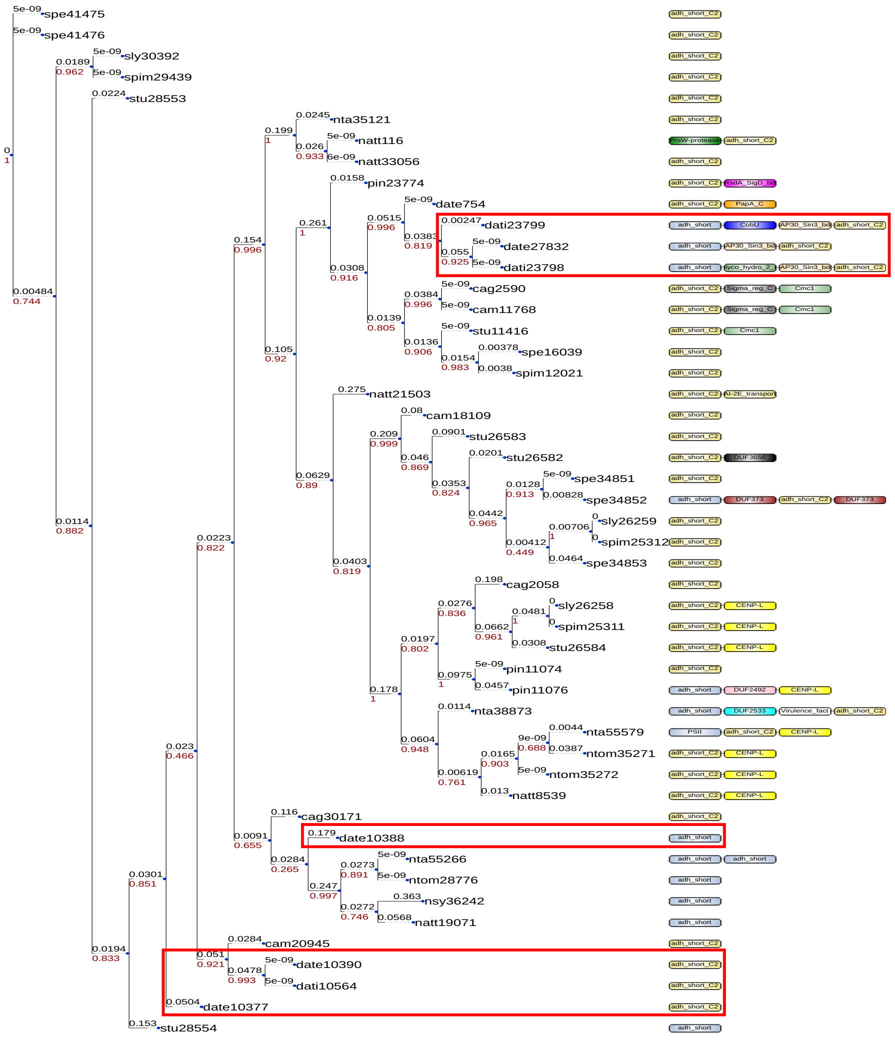

Supplement: Supplementary file 1 — Supplementary Information. [file 41598_2020_79194_MOESM1_ESM.pdf]
